# Ebola virus glycoprotein directly triggers T lymphocyte death despite of the lack of infection

**DOI:** 10.1371/journal.ppat.1006397

**Published:** 2017-05-22

**Authors:** Mathieu Iampietro, Patrick Younan, Andrew Nishida, Mukta Dutta, Ndongala Michel Lubaki, Rodrigo I. Santos, Richard A. Koup, Michael G. Katze, Alexander Bukreyev

**Affiliations:** 1 Department of Pathology, University of Texas Medical Branch, Galveston, Texas, United States of America; 2 Galveston National Laboratory, University of Texas Medical Branch, Galveston, Texas, United States of America; 3 University of Texas Medical Branch, Galveston, Texas, United States of America; 4 Department of Microbiology, University of Washington, Seattle, Washington, United States of America; 5 Immunology Laboratory, Vaccine Research Center, National Institute of Allergy and Infectious Diseases, National Institutes of Health, Bethesda, Maryland, United States of America; 6 Washington National Primate Research Center, Seattle, Washington, United States of America; 7 Department of Microbiology & Immunology, University of Texas Medical Branch, Galveston, Texas, United States of America; St. Jude Children's Research Hospital, UNITED STATES

## Abstract

Fatal outcomes of Ebola virus (EBOV) infections are typically preceded by a ‘sepsis-like’ syndrome and lymphopenia despite T cells being resistant to Ebola infection. The mechanisms that lead to T lymphocytes death remain largely unknown; however, the degree of lymphopenia is highly correlative with fatalities. Here we investigated whether the addition of EBOV or its envelope glycoprotein (GP) to isolated primary human CD4^+^ T cells induced cell death. We observed a significant decrease in cell viability in a GP-dependent manner, which is suggestive of a direct role of GP in T cell death. Using immunoprecipitation assays and flow cytometry, we demonstrate that EBOV directly binds to CD4^+^ T cells through interaction of GP with TLR4. Transcriptome analysis revealed that the addition of EBOV to CD4^+^ T cells results in the significant upregulation of pathways associated with interferon signaling, pattern recognition receptors and intracellular activation of NFκB signaling pathway. Both transcriptome analysis and specific inhibitors allowed identification of apoptosis and necrosis as mechanisms associated with the observed T cell death following exposure to EBOV. The addition of the TLR4 inhibitor CLI-095 significantly reduced CD4^+^ T cell death induced by GP. EBOV stimulation of primary CD4^+^ T cells resulted in a significant increase in secreted TNFα; inhibition of TNFα-mediated signaling events significantly reduced T cell death while inhibitors of both necrosis and apoptosis similarly reduced EBOV-induced T cell death. Lastly, we show that stimulation with EBOV or GP augments monocyte maturation as determined by an overall increase in expression levels of markers of differentiation. Subsequently, the increased rates of cellular differentiation resulted in higher rates of infection further contributing to T cell death. These results demonstrate that GP directly subverts the host’s immune response by increasing the susceptibility of monocytes to EBOV infection and triggering lymphopenia through direct and indirect mechanisms.

## Author summary

The latest outbreak of Ebola virus (EBOV) in West Africa resulted in more than 28,000 human infections including more than 11,000 deaths thus highlighting the necessity for the development of countermeasures. Monocytes and dendritic cells are among the primary targets of EBOV infection; infection of these critical antigen presenting cells contributes to the immune deficiency observed in Ebola virus disease (EVD). In contrast, lymphocytes are resistant to EBOV infection; however, in fatal EVD, pronounced lymphopenia is uniformly observed. Here we report that T lymphocyte cell death in the absence of detectable infection was observed in an EBOV glycoprotein (GP)-dependent manner. Using transcriptome analysis of EBOV-stimulated CD4^+^ T cells we show upregulation of both toll-like receptor 4 (TLR4) and cell death associated pathways. Furthermore, we demonstrate that EBOV increases susceptibility of monocytes to infection by promoting cellular differentiation. Both EBOV-induced monocyte differentiation and cell death of T lymphocytes result from a direct interaction between GP and TLR4. Blocking of TLR4 signaling significantly reduced both EBOV-induced T cell death and infection of monocytes. These data contribute to understanding of the ‘immune paralysis’ during EBOV infections and provide evidence for the development of targeted therapies for the treatment of EVD.

## Introduction

Ebola virus (EBOV) is one of the deadliest pathogens known to exist as evidenced by the latest outbreak in West Africa that resulted in more than 28,000 confirmed and suspected infections including more than 11,000 fatalities [1]. Currently, experimental EBOV candidate vaccines and monoclonal antibody-based therapies are being tested in clinical trials [2, 3]; however, none have yet to be approved for treatment of infected patients. Gaining an in depth understanding of the mechanisms of EBOV’s unparalleled ability to counteract and disrupt the immune response is critical to developing targeted approaches aimed at reducing the pathogenesis directly or indirectly caused by the virus.

A characteristic feature of EBOV infection is the rapid onset of lymphopenia, which is observed in both humans and experimentally infected non-human primates (NHP) [4–11]. Development of lymphopenia is typically observed in EBOV patients that succumb to disease, whereas survivors have been shown to maintain CD3^+^ T lymphocyte populations throughout the course of disease [12, 13]. Strikingly, lymphopenia occurs despite the inability of EBOV to infect lymphocytes [4]. On the other hand, dendritic cells (DCs) and cells derived from monocyte-macrophage lineages are among the primary targets of EBOV infection *in vivo* [11, 14]. EBOV infection of these cells results in their aberrant activation [15–18] and induction of Fas and tumor necrosis factor related cell death inducing factor (TRAIL) pathways [19, 20]. We and others recently demonstrated that the lack of proper maturation of these critical antigen presenting cells (APCs) results in a limited activation of antigen-specific T lymphocytes [21, 22] further contributing to the deficient adaptive immune response. In addition, infection of monocyte-macrophage lineage may also contribute to EBOV-associated pathogenesis by several mechanisms including the release of inflammatory mediators, which may contribute to their apoptosis and necrosis [23].

The only EBOV envelope glycoprotein (GP) was shown to bind and activate the TLR4 signaling pathway [24]. TLR4 is known to trigger both apoptotic and necrotic pathways *via* direct and/or indirect activation of infected cells or bystander cells, which may contribute to these inflammatory mechanisms [25]. Lastly, TLR4 stimulation of cells of the monocyte/macrophage lineage and DCs results in their activation and/or differentiation [26, 27]. Cellular differentiation may further contribute to the release of inflammatory mediators associated with the onset of a cytokine storm, which is a characteristic feature of EBOV infection [28–30].

Since lymphocytes are resistant to EBOV infection, the mechanisms causing lymphopenia during EBOV infection remain largely unknown. Hence, the primary goal of this study was to examine whether EBOV directly stimulates T lymphocytes and determine the direct and indirect role of TLR4 in mediating T cell death in the pathogenesis of EBOV infection.

## Results

### EBOV directly induces cell death of T lymphocytes

**Fig 1:**
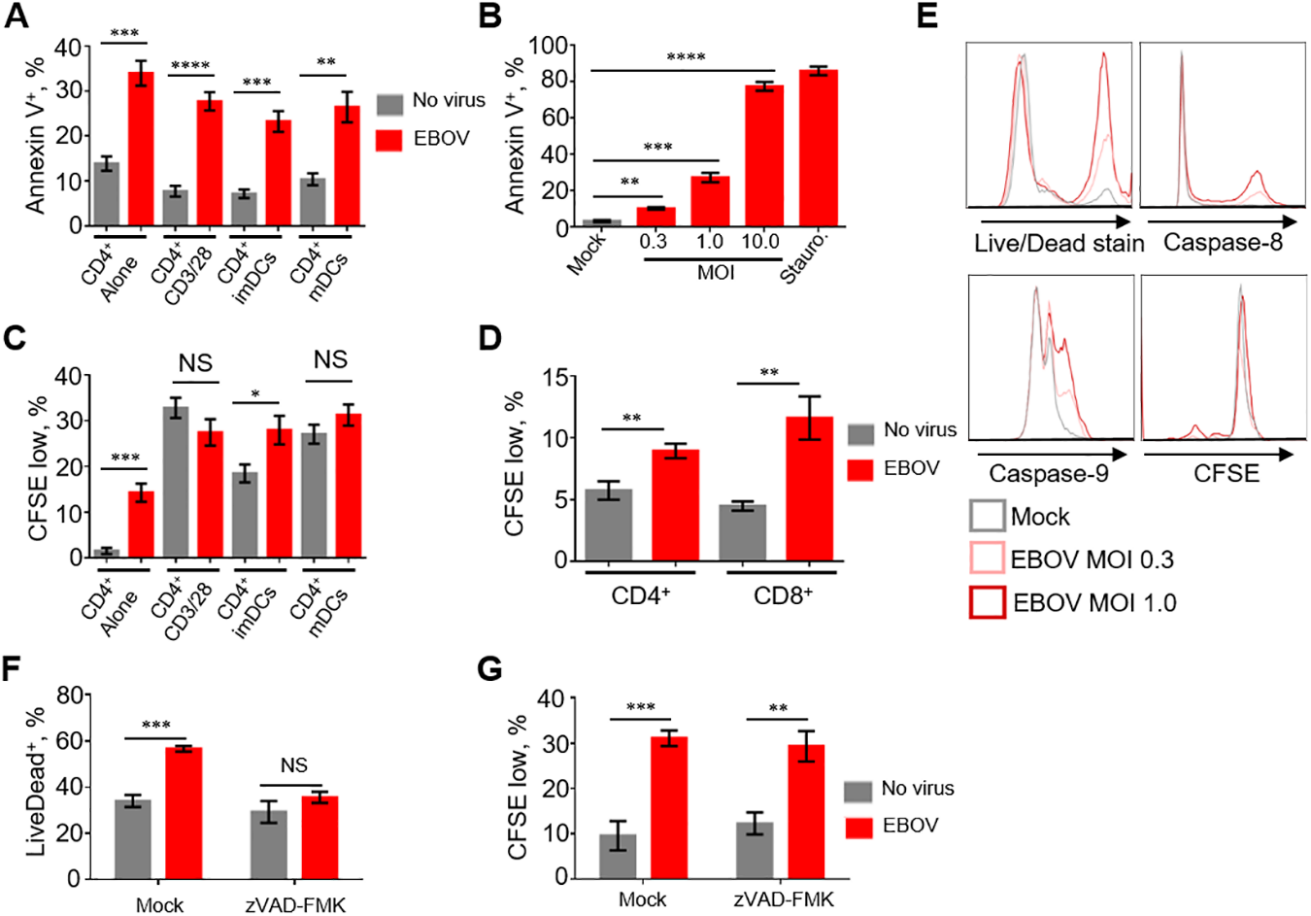
EBOV induces cell death of T lymphocytes. **A.** Percentages of annexin-V^+^ cells in preparations of CD4^+^ T lymphocytes cultured alone, activated overnight with CD3/CD28 beads, or co-cultured with imDCs or mDCs in the absence (mock) and presence of EBOV (MOI 0.3 PFU/cell) for 7 days. **B**. Percentages of annexin-V^+^ CD4^+^ T cells following incubation of with EBOV at MOI of 0.3, 1.0 or 10.0 PFU/cell for 4 days. Staurosporine (1 μM final concentration) treatment was used as a positive control for cell death. **C**. Percentages of CFSE_low_ (divided) CD4^+^ T lymphocytes treated as indicated in panel A. **D**. Percentages of CFSE_low_ (divided) CD4^+^ and CD8^+^ T lymphocytes cultured in the presence of EBOV for 4 days at MOI 0.3 PFU/cell. **E**. Flow cytometry analysis of CD4^+^ T lymphocytes cultured with EBOV for 4 days at MOI of 0.3 or 1.0 PFU/cell: dead (Live/Dead^+^) cells, cells positive for activated caspase-8 and activated caspase-9, and CFSE_low_ (divided) cells. **F, G**. Percentages of dead (Live/Dead^+^) cells and proliferation of isolated CD4^+^ T cells exposed to EBOV for 4 days in the presence of the apoptosis inhibitor zVAD-FMK. Data are representative of triplicate samples from one of 7 independent donors. * *P*<0.05, ** *P*<0.01, *** *P*<0.001, **** *P*<0.0001, n.s., non-significant (Student T-test).

As lymphopenia is a common feature observed in fatal cases following EBOV infection [4–11], we first sought to determine if a direct interaction of EBOV with DCs can cause T cell death. To visualize the infection, we used a recombinant EBOV expressing enhanced green fluorescent protein (GFP) from an added transcriptional cassette (EBOV-GFP); this virus replicates in cultured cells at the same level as wt EBOV [31]. Human monocyte-derived DCs and autologous T lymphocytes were co-cultured with EBOV-GFP for 7 days, and the percentages of apoptotic CD4^+^ or CD8^+^ T cells were determined by annexin-V staining. We also included primary lymphocyte cultures in which highly purified naïve or CD3/CD28 activated CD4^+^ T cells were exposed directly to EBOV-GFP in the absence of DCs or monocytes. Culturing of CD4^+^ T cells in the presence of EBOV-infected mature (by adding TNFα) or immature DCs resulted in a significant increase of apoptotic cells (Fig 1A). Unexpectedly, the highest level of cell death was observed when isolated CD4^+^ T cells were cultured alone in EBOV-containing medium and this effect was observed to be dose-dependent (Fig 1B, S1A and S1B Fig). Similar results were observed with isolated CD8^+^ T cells (S1C Fig). We also observed an elevated level of proliferation of CD4^+^ T cells cultured with EBOV alone or with EBOV-infected immature DCs, but not CD3/CD28 bead-stimulated or mature DCs (Fig 1C). Similarly, EBOV induced proliferation of CD8^+^ T cells (Fig 1D, S1D Fig). These data suggest that EBOV is capable of inducing non-specific proliferation of lymphocytes. Five days after addition of EBOV-GFP, we detected a dose-dependent increase in the percentages of dead CD4^+^ T cells or cells positive for activated caspase-8 and caspase-9, as well as proliferated cells (Fig 1E). The addition of the inhibitor of apoptosis z-VAD-FMK significantly reduced cell death associated with EBOV; however, proliferation remained unabated (Fig 1F and 1G). Similarly, the addition of the pro-survival cytokines IL-7 and IL-15 significantly reduced the percentages of apoptotic CD4^+^ and CD8^+^ T cells cultured in the presence of EBOV (S1E and S1F Fig). The infectivity of EBOV incubated under the experimental conditions used for these studies was determined by daily collection of aliquots from cell-free medium, their flash freezing, infection of Vero E6 cells and flow cytometry analysis of the percentages of GFP^+^ cells. A moderate reduction of infectivity not exceeding 32% was detected on days 1–3, followed by ~66% reduction of infectivity on days 4–6 (data now shown). These data demonstrate that EBOV directly triggers apoptotic death of T cells, despite the lack of their infection.

### Cell death of T lymphocytes is triggered by direct binding of GP

**Fig 2:**
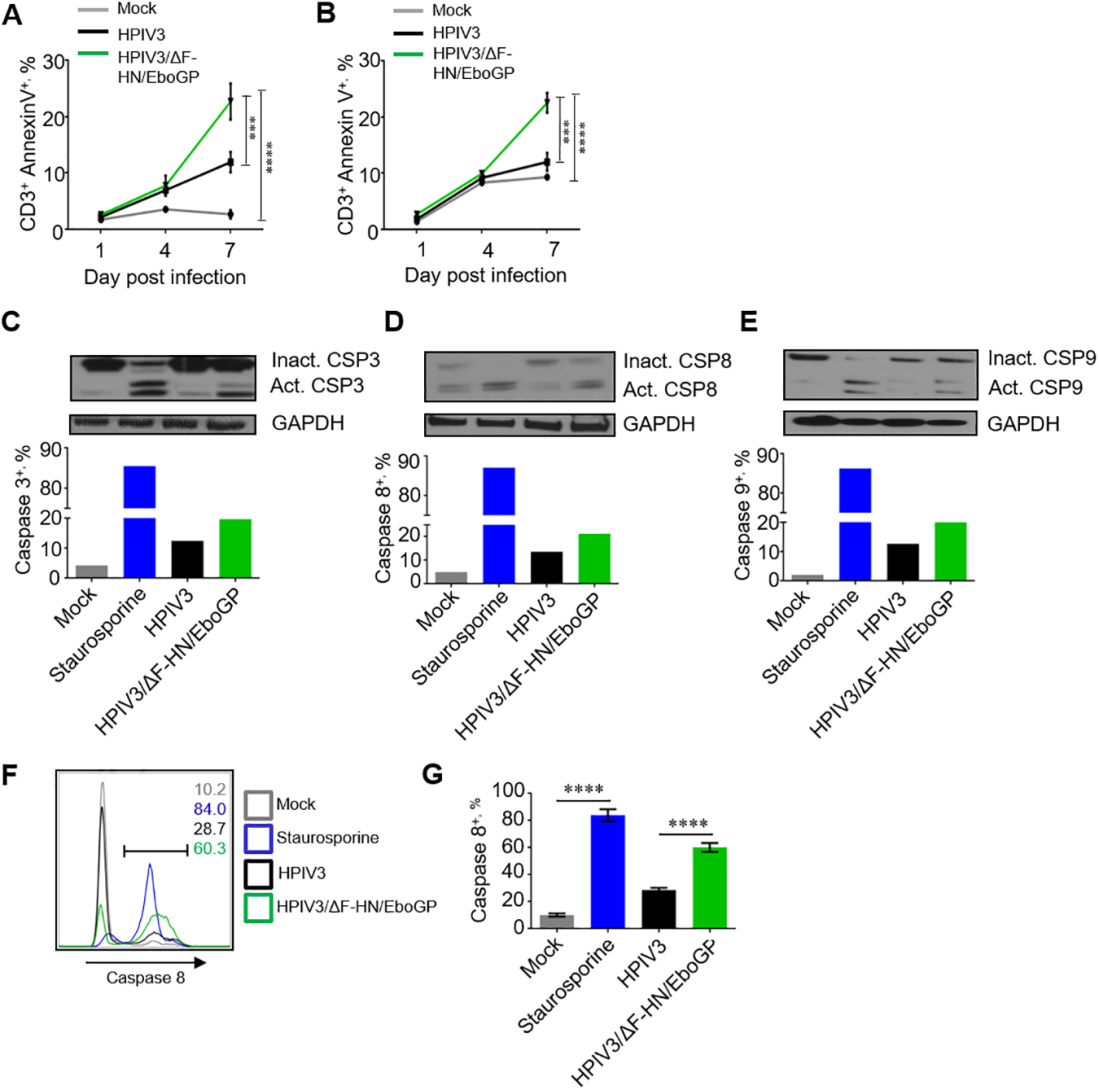
Cell death of T lymphocytes is triggered by direct binding of GP. **A.** Percentages of annexin V^+^ CD3^+^ T cells in PBMCs incubated with HPIV3/ΔF-HN/EboGP or HPIV3 for 1, 4 and 7 days determined by flow cytometry. Mean values ±SE based on 3 donors. **B.** Percentages of annexin-V^+^ isolated primary CD3^+^ T cells incubated with HPIV3/ΔF-HN/EboGP or HPIV3 for 1, 4 or 7 days at 37°C determined by flow cytometry. Mean values ±SE based on 3 donors. A, B, Two-Way ANOVA followed by a Tukey’s multiple comparisons test, *P* values are indicated in Fig 1**C,** 1**D** and 1**E**. Activation of caspases in Jurkat cells incubated with HPIV3 or HPIV3/ΔF-HN/EboGP for 7 days or stimulated with staurosporine for 6 h determined by Western blotting: non-activated and activated caspases 3 (**C**), 8 (**D**) and 9 (**E**). Amounts of active caspases as percentages of total (active and inactive) caspases. Panels C, D and E are one set of representative data from two independent experiments. **F, G.** Flow cytometry analysis of activated caspase 8 in Jurkat cells incubated with HPIV3/ΔF-HN/EboGP or HPIV3 for 7 days or stimulated with staurosporine (control) for 6 h: representative primary data with percentages of positive populations indicated for each treatment (F) and mean percentages of caspase-8^+^ cells ±SE based on triplicate samples from one of three independent experiments. *P* values * *P*<0.05, ** *P*<0.01, *** *P*<0.001, **** *P*<0.0001 (Student T-test) (G).

We next tested if death of T lymphocytes exposed to EBOV is caused by binding of GP. We used the chimeric parainfluenza virus type 3 in which its envelope proteins HN and F are replaced with EBOV GP (HPIV3/ΔF-HN/EboGP); the structure of GP at the surface of this chimeric virus has been shown to be identical to that found on the surface of EBOV particles [32]. Total PBMCs or purified CD3^+^ T lymphocytes were cultured for 1, 4 or 7 days in the presence of HPIV3 or HPIV3/ΔF-HN/EboGP and stained with annexin-V. Cultivation with HPIV3/ΔF-HN/EboGP resulted in a significant increase compared to HPIV3 in the percentages of dead (annexin V^+^) cells in gated CD3^+^ T cells in total PBMCs (Fig 2A) and in purified CD3^+^ T cells directly exposed to EBOV (Fig 2B). We also sought to evaluate cell death and the role of EBOV GP by using the chimeric vesicular stomatitis virus VSVΔG/ZEBOVGP in which the sole envelope protein G was replaced with EBOV GP [33]. A 4 day-long incubation of SupT1, a lymphoblastoid CD4^+^ T cell line, with this virus resulted in a dose-dependent cell death (S2A and S2B Fig). The findings suggest that EBOV-induced apoptosis of T cells is directly associated with EBOV GP.

We next determined if exposure of lymphocytes to GP activates apoptotic pathways. Jurkat cells were cultured in presence of HPIV3/ΔF-HN/EboGP, HPIV3 or staurosporine, which is a strong inducer of apoptosis, for 7 days. We found that culturing with HPIV3/ΔF-HN/EboGP or staurosporine caused a strong increase in the levels of activated caspases 3, 8 and 9, which were higher than that in cells treated with HPIV3 or mock treated cells (Fig 2C–2E). To characterize caspase activation at a single-cell level, the cultured Jurkat cells were also analyzed for activated caspase 8 by flow cytometry; we found that culturing with HPIV3/ΔF-HN/EboGP and HPIV3 resulted in 60.3±1.9% and 28.7±0.9% of cells, respectively, positive for active caspase 8 (Fig 2F and 2G). Taken together, this data indicates that EBOV triggers cell death pathways through GP-dependent mechanisms.

### GP mediates binding of EBOV to T lymphocytes through TLR4

**Fig 3:**
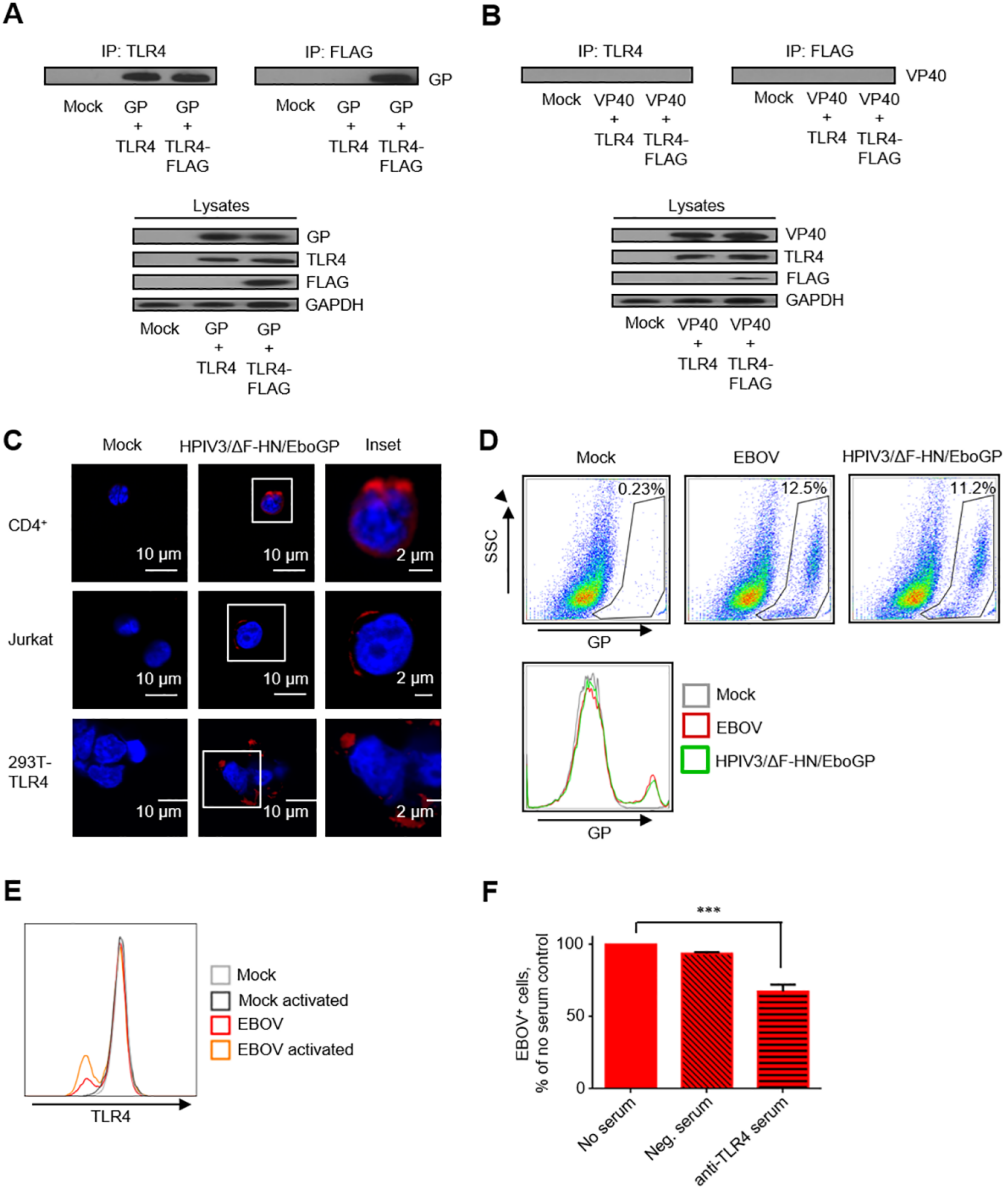
EBOV GP mediates binding to T lymphocytes through TLR4. **A**, **B**. Western blotting analysis of immunoprecipitation of TLR4 or TLR-4 FLAG with EBOV GP (A) or EBOV VP40 (B). Representative data from one of two independent experiments. **C.** Confocal microscopy of HPIV3/ΔF-HN/EboGP bound to primary CD4^+^ T lymphocytes, Jurkat cells and 293T-TLR4 cells. Insets show the formation of plasma membrane associated GP-positive puncti. **D.** Flow cytometry analysis demonstrating the binding of EBOV and HPIV3/ΔF-HN/EboGP to SupT1 T cells. **E.** Flow cytometry analysis of TLR4 expression by isolated CD4^+^ T cells. T cells were activated with CD3/CD28 beads and then cultured with EBOV. Results are representative of 3 donors. **F**. Inhibition of EBOV binding to SupT1 cells by anti-TLR4 serum: % of no serum control. Mean values ±SE based on triplicate samples of one of two independent experiments, *** P<0.001 (Student T-test).

Since T lymphocytes express TLR4 [34], we hypothesized that the virus can attach to T lymphocytes. Previous reports have indicated that EBOV GP binds to and activates TLR4 signaling in DCs [24, 35]. We confirmed binding following transfection of 293T human embryo kidney cells with plasmids expressing EBOV GP, VP40 and/or TLR4/TLR4-FLAG. EBOV GP, but not VP40, efficiently co-precipitated with anti-TLR4 antibodies, suggesting that GP specifically interacts with TLR4 (Fig 3A and 3B). To determine if GP mediates binding to T lymphocytes, we used confocal microscopy and flow cytometry; to distinguish the role of GP as opposed to whole EBOV, we utilized HPIV3/ΔF-HN/EboGP. As expected, both confocal microscopy and flow cytometry demonstrated direct binding of EBOV and HPIV3/ΔF-HN/EboGP to control 293T cells expressing TLR4 from a transfected plasmid [36], as wild-type 293T cells do not express TLR4 [37]. Importantly, binding of EBOV and HPIV3/ΔF-HN/EboGP to purified human CD4^+^ T cells, Jurkat cells and SupT1 cells previously shown to possess a strong TLR4 signaling pathway following activation [38] was observed (Fig 3C and 3D). EBOV stimulation of naïve and CD3/CD28-bead activated CD4^+^ T cells reduced the relative density of TLR4 on cell surface (Fig 3E), suggesting that TLR4 may be internalized. Moreover, blocking of TLR4 with polyclonal anti-TLR4 antibodies significantly reduced binding of EBOV to SupT1 T cells (Fig 3F). These data demonstrate that EBOV GP binds to T cells *via* TLR4 resulting in stimulation of cells despite the lack of infection.

### EBOV GP induced cell death of T lymphocytes is triggered *via* TLR4 activation

**Fig 4:**
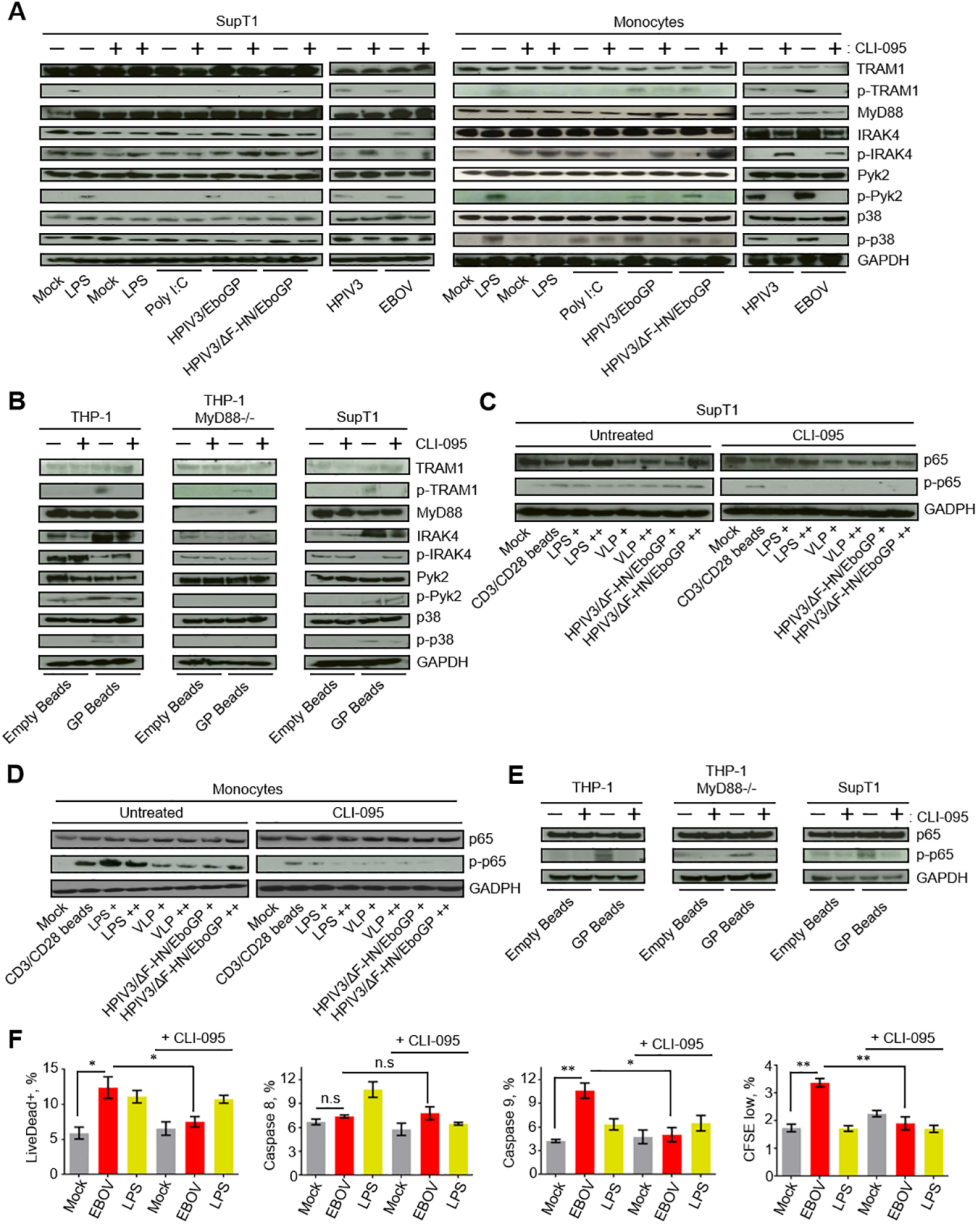
EBOV GP activates the TLR4 pathway that leads to T cell death. **A, B.** Western blot analysis of proteins involved in TLR4 signaling pathway TRAM1, p-TRAM1, MyD88, IRAK4, p-IRAK4, Pyk2, p-Pyk2, p38, p-p38 in SupT1 cells and monocytes (A) or THP-1, THP-1 MyD88-/- and SupT1 cells (B) following stimulations with LPS, poly I:C, HPIV3/EboGP, HPIV3/ΔF-HN/EboGP, HPIV3 or EBOV (A) or empty beads or EBOV GP beads (B) in the presence or absence of the TLR4 inhibitor CLI-095. **C**, **D**, **E**. Western blot analysis of p65 phosphorylation in SupT1 cells (C), monocytes (D) or THP-1, THP-1 MyD88-/- and SupT1 cells (E) following stimulation with CD3/CD28 beads, LPS (+, 100 ng/ml, ++; 500 ng/ml), VLP (+, 100 μl; ++, 250 μl) and HPIV3/ΔF-HN/EboGP (+, MOI 0.1 PFU/cell; ++, MOI 1 PFU/cell), empty beads or EBOV GP beads, as indicated, with or without CLI-095. Western blots in panels A-E are representative of two independent experiments. **F**. Percentages of dead (Live/Dead^+^) cells, cell positive for caspase 8 and 9 and proliferated CD4^+^ T lymphocytes following a 4 day-long incubation with EBOV or LPS with or without CLI-095. Mean values ±SE based on triplicates from one of two independent experiments with *P* values * *P*<0.05, ** *P*<0.01, n.s., non-significant (Student T-test).

A functional TLR4 response in primary T lymphocytes has been demonstrated [39]; we therefore sought to determine if TLR4 signaling was the initial trigger of primary CD4^+^ T cells death exposed to EBOV GP. We previously confirmed that EBOV binds to SupT1 cells (Fig 3D). The relative binding efficiency of EBOV appeared to be similar to that observed on primary CD4^+^ T cells and Jurkat cells. SupT1 cells or monocytes, which were used as control cells susceptible to EBOV infection, were cultured in the presence of EBOV, the natural TLR4 ligand lipopolysaccharide **(**LPS) [40], HPIV3, its derivative expressing EBOV GP from an added transcriptional cassette HPIV3/EboGP [41], HPIV3/ΔF-HN/EboGP described above, or the TLR3 agonist polyI:C, which was used as a control for TLR specificity. Cells were cultured in the presence or absence of the TLR-4 inhibitor CLI-095 to determine the role of TLR4 signaling. Activation of TLR4 signaling cascade was examined by analysis of the phosphorylation state of TLR4 adapter proteins p-TRAM1, phosphorylated following endosomal translocation of TLR4 and activating MyD88-independent pathway [42], dephosphorylated IRAK4, p-Pyk2 or p-p38. The data demonstrated activation of TLR4 signaling following stimulation with EBOV or its GP protein in both SupT1 cells and monocytes with a reduction in TLR4 signaling being observed when cells were cultured with CLI-095 (Fig 4A). To more specifically demonstrate that EBOV stimulated TLR4 through its glycoprotein GP, we evaluated the capacity of recombinant GP-bound beads or empty beads to activate both the TRAM1 and MyD88-dependent pathways using THP-1, THP-1 MyD88^-/-^ and SupT1 cells (Fig 4B, S3A Fig). While in THP-1 and SupT1 cells GP beads were able to activate TLR4 signaling, in THP-1 MyD88^-/-^ cells they were unable to trigger dephosphorylation of IRAK4, phosphorylation of Pyk2 or p38, while phosphorylation of TRAM1 still occurred. The addition of CLI-095, which blocks both the TRAM1 and MyD88 pathways, significantly reduced TLR4-associated signal transduction. These data demonstrate that EBOV GP induces TLR4 signaling in CD4^+^ T cells by triggering both MyD88-dependent and MyD88-independent pathways.

We next examined activation of NFκB, a downstream transcription factor known to be activated following TLR4 stimulation [36], by stimulating SupT1 cells, monocytes, THP-1 and THP-1 MyD88^-/-^ cells with GP delivered by HPIV3/ΔF-HN/EboGP, virus-like particles (VLPs) or GP beads (Fig 4C–4E). Cell lysates were examined for the phosphorylation status of the p65 subunit of NFκB, which plays a major role in immune and inflammatory responses and whose phosphorylation is indicative of NFκB signaling activation [43]. Peak phospho-p65 (p-p65) was detected 2 h after stimulation (S3B Fig). Stimulation of SupT1 cells, monocytes, THP-1 and THP-1 MyD88 -/- cells with EBOV, HPIV3/ΔF-HN/EboGP, VLPs, GP beads or LPS, as well as control CD3/CD28 activation beads, resulted in a marked increase in phosphorylated p65 (Fig 4C–4E). Treatment of cells with CLI-095 for 1 h prior to stimulation reduced the levels of phosphorylated p65 associated with EBOV, HPIV3/ΔF-HN/EboGP, VLPs, GP beads or LPS stimulation. As expected, CLI-095 did not affect phosphorylation of p65 in control CD3/CD28 activated cells (Fig 4C and 4D). These findings further confirm that EBOV stimulation results in TLR4-mediated signal transduction in primary CD4^+^ T cells by both MyD88-dependent and MyD88-independent signaling pathways.

Next, we determined the role of TLR4 signaling in the previously observed EBOV GP-mediated T lymphocytes death. Purified primary CD4^+^ T cells were cultured in the presence of EBOV or LPS with or without the TLR4 inhibitor CLI-095. EBOV and LPS induced similar rates of cell death; however, cell death associated with EBOV induced extensive activation of caspase 9, whereas LPS primarily triggered activated caspase 8 (Fig 4F, S3C Fig). Interestingly, the rate of proliferation was increased by EBOV but not by LPS. Importantly, inhibition of TLR4 significantly reduced the percentages of dead cells with a correlative decrease in activated caspase 9 being observed in the presence of CLI-095 (Fig 4F, S3C Fig). Taken together, these findings indicate that death of CD4^+^ T cells exposed to EBOV is associated with both intrinsic and extrinsic pathways.

### Exposure of T lymphocytes to EBOV triggers multiple pathways associated with cell death

Based on these findings, we conducted a series of experiments aimed at determining the mechanisms by which EBOV induces T lymphocyte cell death. First, transcriptome analysis was utilized to determine the global response of CD4^+^ T cells to EBOV stimulation. Deep sequencing was performed on RNA samples extracted from highly purified CD4^+^ T cells cultured in medium alone, with EBOV or LPS at 24 and 96 h. Differential expression (DE) analysis comparing EBOV- and mock-infected samples at 24 h resulted in 2,591 DE genes using a 1.5 fold change cutoff and an adjusted *p*-value of 0.05 as criteria. A significant portion of these DE genes were related to cell death and innate immunity sensing. Specifically, exposure of T lymphocytes to EBOV resulted in 265 DE genes related to pathways specific for apoptosis, necrosis and TLR4 signaling (S4A–S4C Fig). These results are consistent with the observations in Fig 1E demonstrating increased caspase-dependent cell death and TLR4 activation following exposure of T cells to EBOV. Overall, a global transcriptome profile of CD4^+^ T cells cultured in the presence of EBOV or LPS resulted in a much greater number of upregulated genes than downregulated genes and a greater number of differentially regulated genes involved in necrosis than apoptosis (S4A–S4C Fig). In addition, the number of genes whose expression was differentially regulated was remarkably similar between EBOV and LPS (Table 1). However, the pattern of gene response to EBOV was clearly distinct of that induced by LPS (S4A Fig). As indicated in the heat map and gene network in S4A and S4B Fig, TLR4 activation is associated with both apoptotic and necrotic pathways. Induction of multiple cell death pathways by EBOV explains why this virus but not HPIV3 strongly induces cell death (Fig 2A and 2B) even though both viruses induce TLR4 (Fig 4A).

**Table 1:** EBOV-induced gene expression changes related to different cell death mechanisms/pathways.

Stimulation, day	Apoptosis	Necrosis	TLR	Cell death
Increased expression, number of genes				
EBOV d.1	20	62	72	163
EBOV d.4	18	49	58	41
LPS d.1	18	54	63	143
LPS d.4	15	41	51	100
Reduced expression, number of genes				
EBOV d.1	4	13	19	40
EBOV d.4	6	26	33	79
LPS d.1	6	21	28	60
LPS d.4	9	34	40	103
Increased expression, top 20% genes				
EBOV d.1	3	18	18	41
EBOV d.4	4	26	28	30
LPS d.1	4	17	17	38
LPS d.4	4	21	24	33
Reduced expression, top 20% genes				
EBOV d.1	3	13	19	39
EBOV d.4	4	11	16	41
LPS d.1	2	12	18	39
LPS d.4	3	18	21	40

The numbers of upregulated and downregulated genes from S4A Fig selected according the four biological functions: the total number of genes and the top 20% by the change in the level of expression.

**Fig 5:**
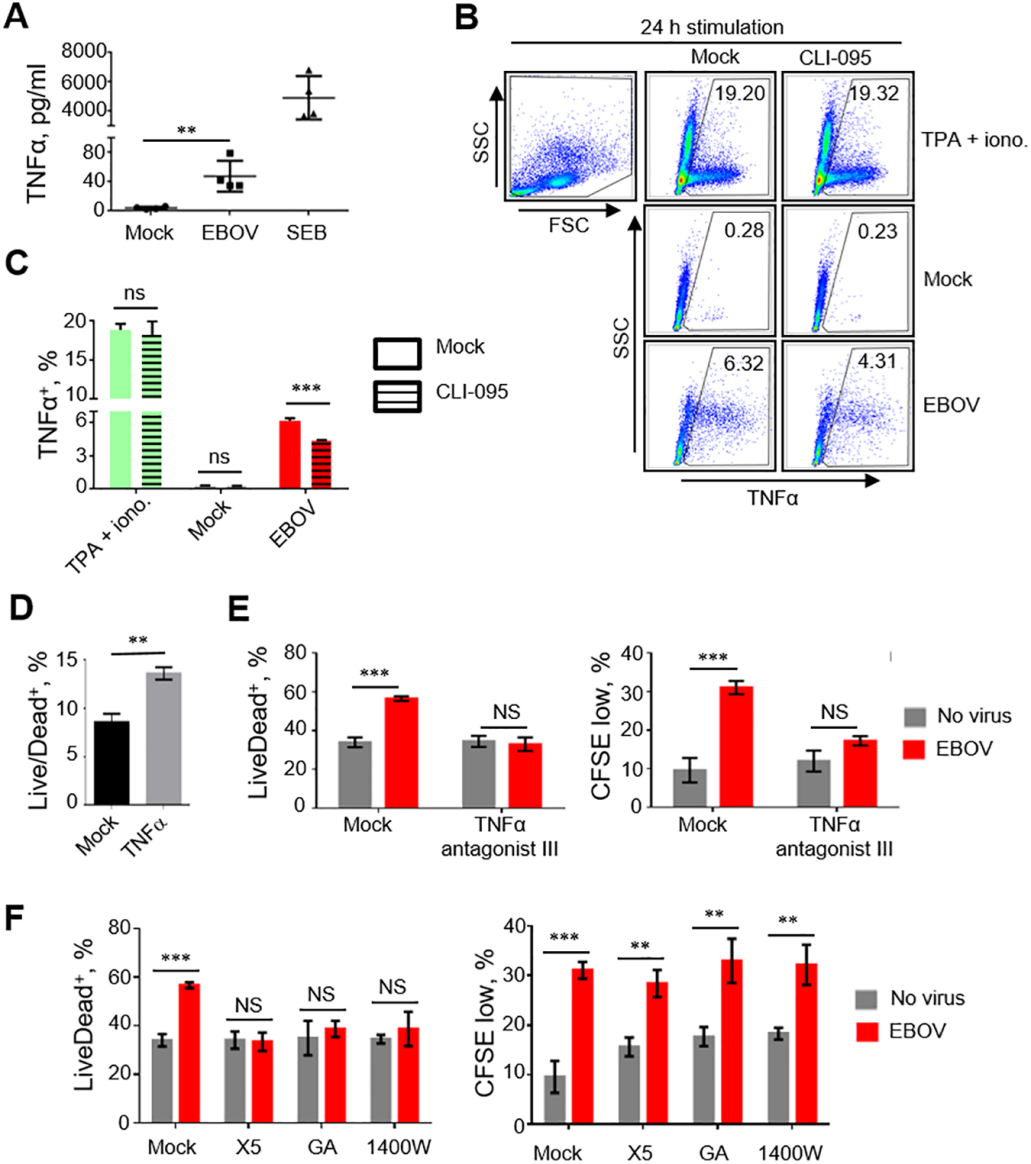
Mechanisms of cell death caused by EBOV. **A.** Concentrations of TNFα in medium of purified CD4^+^ T cells cultured with EBOV or SEB for 4 days. Mean values ±SE based on 4 donors analyzed in duplicates. *P* values * *P*<0.05, ** *P*<0.01, *** *P*<0.001, **** *P*<0.0001 (Student T-test). **B**, **C**. Flow cytometry analysis of the percentages of TNFα^+^ SupT1 cells cultured with medium (mock), TPA/ionomycin or EBOV, mean values ±SE based on triplicate samples from one of two independent experiments shown (C). **D**. Percentages of Live/Dead^+^ SupT1 cells following daily additions of TNFα at 80 pg/ml for 4 days: mean values ±SE based triplicate samples from one of two independent experiments. **E.** Effects of TNFα antagonist III on CD4^+^ T cell death induced by EBOV at 4 days post infection: percentages of Live/Dead^+^ cells (left panel) and proliferated cells (right panel). Mean values ±SE based on triplicate samples from one of 3 independent experiments. **F.** Percentages of dead (Live/Dead^+^) cells (left panel) and proliferation (right panel) in the presence of necrosis inhibitors NecroX5 (X5), geldanamycin (GA) or N-(3-aminomethyl)benzylacetamindine (1400W) added immediately prior to the addition of EBOV at 4 days post infection. Mean values ±SE based on triplicate samples from one of 3 independent experiments. *P* values for panels E, F: ** *P*<0.01, *** *P*<0.001, n.s., non-significant (Student T-test).

We next investigated the contributing roles of each cell death pathway on EBOV-mediated T cell death. Due to the essential role of TNFα as an immune modulator following TLR4 activation and its role as an inducer of both apoptotic and necrotic pathways [44], we hypothesized that TNFα inhibition may reverse cell death induced by EBOV. We first determined the capacity of isolated CD4^+^ T cells to release TNFα when cultured with EBOV. Indeed, a 96 h-long incubation of cells with EBOV or a positive control staphylococcal enterotoxin B (SEB) resulted in increased levels of TNFα, 47.5±10.6 and 4,913±738 pg/ml, respectively, which were significantly greater than in mock-treated samples, 4.0±0.8 pg/ml (Fig 5A). To confirm that TNFα expression is TLR4-dependent, we treated or mock-treated SupT1 T cells with CLI-095 or anti-TLR4 neutralizing antibodies, incubated with EBOV or 12-*O*-tetradecanoylphorbol-13-acetate (TPA) /ionomycin for 24 h and analyzed for TNFα or IFNγ, as an additional marker of T cell activation. Our results demonstrated an increase in intracellular TNFα^+^ SupT1 cells following cultivation with TPA/ionomycin (19.2% of TNFα^+^ cells) and EBOV (6.3%) when compared to mock (0.3%) (Fig 5B and 5C). Treatment of cells with CLI-095 reduced the percentages of TNFα^+^ cells only when they were infected with EBOV, to 4.3%. Treatment of SupT1 cells with TPA/ionomycin or EBOV resulted in an increase in percentages of TNFα^+^ and IFNγ^+^ cells (17.2% and 24.5% for TPA/ionomycin and 8.9% and 18.1% for EBOV, respectively) when compared to mock (3.6% and 6.0%, respectively) (S5A and S5B Fig). Pre-incubation of cells with anti-TLR4 reduced the percentages of TNFα^+^ and IFNγ^+^ cells in EBOV treated cells by 22.4% and 27.7%, respectively. Daily treatment of cells with TNFα at 80 pg/ml significantly increased the percentages of dead cells (Fig 5D), while the addition of an inhibitor of TNFα, TNFα antagonist III, which blocks TNFα receptor-adapter interactions and prevents downstream signaling [45], completely reversed TNFα-associated cell death (Fig 5E, left panel). Interestingly, treatment with TNFα antagonist III also reduced cellular proliferation suggesting that TNFα may promote cell activation (Fig 5E, right panel). Transcriptome analysis further supported these findings, as 24 h-long exposure of CD4^+^ T cells to EBOV resulted in a 5.1±1.6 fold increase in the levels of TNFα transcripts (p<0.05, Student’s T-test, based on log_2_ values) (S4A Fig). As TNFα is also involved in necrosis, we further confirmed the role of necrotic pathways in the observed EBOV-mediated induction of cell death using necrosis inhibitors. The addition of Necro X5, geldanamycin or 1400W drastically reduced CD4^+^ T cells death following exposure to EBOV without affecting cell proliferation (Fig 5F) suggesting induction of necrosis. These findings demonstrate that T lymphocyte death induced by EBOV is associated with the engagement of TLR4 and subsequent production of TNFα which lead to induction of both apoptotic and necrotic pathways. Therefore, targeting of TLR4 or TNFα signaling cascades may provide therapeutic intervention strategies against EBOV infections.

### EBOV GP activates TLR4 and promotes differentiation of monocytes resulting in an increased susceptibility to infection

As noted, EBOV disease is characterized by infection of multiple types of cells including DCs and cells of the monocyte/macrophage lineage, a high and uncontrolled inflammatory response and depletion of T cells. Previous findings demonstrated that monocyte activation and differentiation increases their susceptibility to EBOV [46]. Furthermore, stimulation of TLR4 by EBOV GP was demonstrated to activate 293T cells [24] and DCs [35]. We hypothesized that the increased susceptibility of monocytes to EBOV infection is related to their differentiation caused by EBOV GP triggering TLR4 signaling. To test the hypothesis, we used THP-1 cells; differentiation was characterized by analysis of activation/differentiation markers CD14, CD11b [47] and CD68 [48] by flow cytometry.

**Fig 6:**
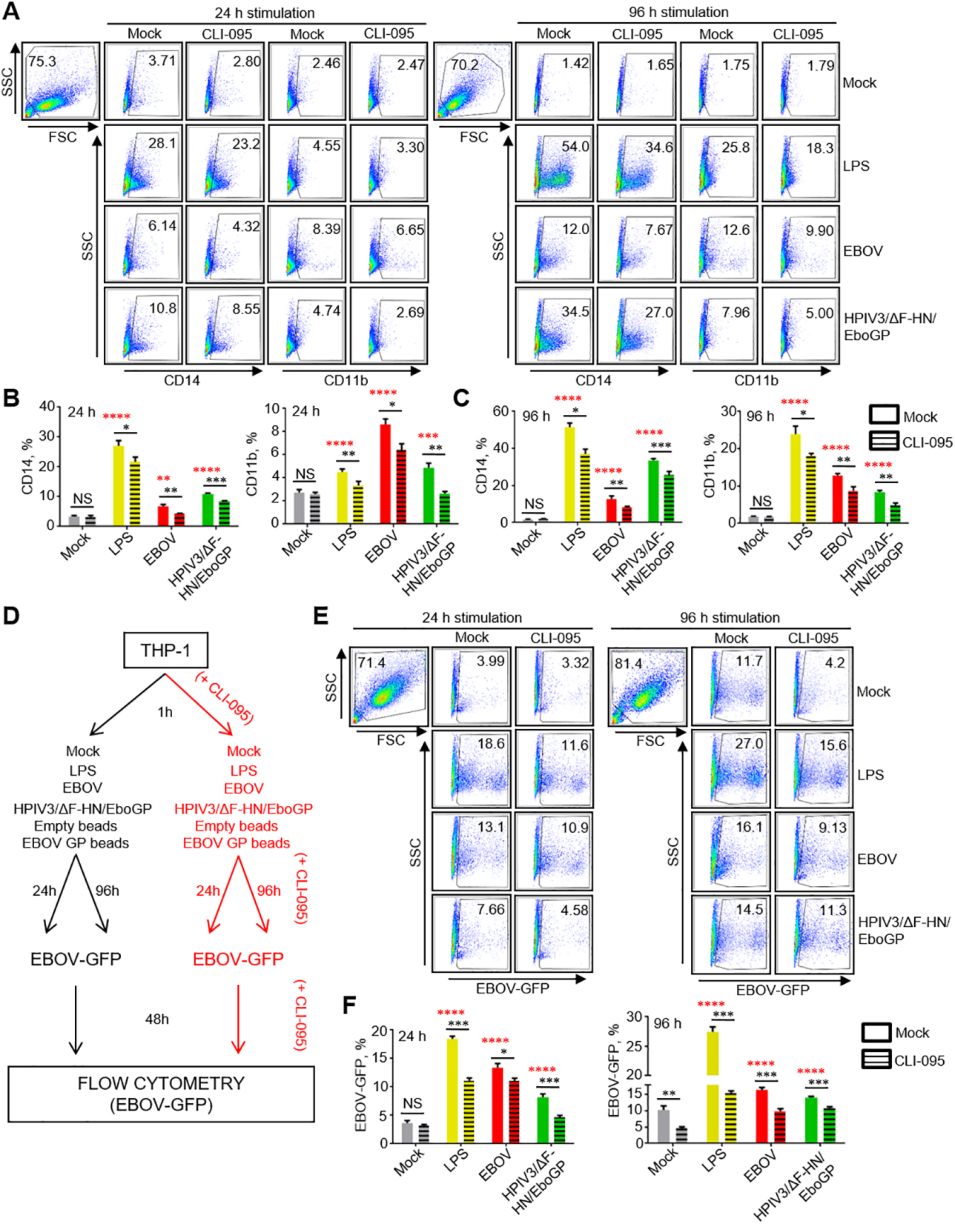
EBOV promotes differentiation and infection of THP-1 cells through TLR4 activation. **A-C,** Effects of EBOV or GP on differentiation of THP-1 cells. Cells were mock-treated or treated with CLI-095, cultured with medium (mock), LPS, EBOV (no GFP) or HPIV3/ΔF-HN/EboGP for 24 or 96 h and analyzed for activation markers CD14 and CD11b by flow cytometry. Representative primary data (A) and mean values ±SE for 24 h (B) and 96 h based on triplicate samples from one of two independent experiments. (C). **D.** The experimental design to evaluate the role of GP in EBOV infection of THP-1 cells. **E, F.** Infection of THP-1 cells following stimulation with LPS, EBOV-no GFP or HPIV3/ΔF-HN/EboGP for 24 or 96 h and infection with EBOV-GFP analyzed by flow cytometry: representative primary flow cytometry data showing expression of GFP (E) and mean values ±SE based on triplicate samples from one of two independent experiments with *P* values (Two-Way ANOVA followed by a Tukey’s multiple comparison test and multiple T-tests), * *P*<0.05, ** *P*<0.01, *** *P*<0.001, **** P<0.0001, n.s., non-significant, black asterisks, difference to CLI-095-treated cells, red asterisks, difference to mock-stimulated cells (F).

First we tested if GP-mediated activation of TLR4 can result in differentiation of THP-1 cells. As TLR4-mediated stimulation of monocytes typically leads to differentiation into macrophages [26], THP-1 cells were cultured with LPS, EBOV, HPIV3/ΔF-HN/EboGP and GP beads in the presence or absence of CLI-095 for 24 h or 96 h (S6A Fig). Our results demonstrated a significant increase in the levels of markers of differentiation at both time points compared to mock-stimulated or empty beads-stimulated cells (Fig 6A–6C, S6B and S6C Fig, S7A–S7H Fig). Higher levels of differentiation were observed at 96 h compared to 24 h post-infection (S6D–S6F Fig, S7I–S7K Fig), which was consistent with the increase of infected cells as determined by the percentage of GFP^+^ cells (S6G Fig, S8A–S8D Fig). CLI-095 treatment significantly reduced the expression of the differentiation markers, which was more pronounced at 96 h, clearly implicating the role of TLR4 in EBOV-induced differentiation of THP-1 (Fig 6A–6C and S6B and S6C Fig). Specifically, in cells treated with LPS, EBOV, HPIV3/ΔF-HN/EboGP and GP beads, we detected a reduction in the percentages of cells positive for CD14 and CD11b. We next determined the relative rates of infection of THP-1 cells following TLR4-mediated differentiation. Following culture in the presence of LPS, EBOV, HPIV3/ΔF-HN/EboGP or GP beads for 24 or 96 h with or without CLI-095, cells were infected for 48 h with EBOV-GFP (Fig 6D). Consistent with the data on the effects of LPS, EBOV, HPIV3/ΔF-HN/EboGP and GP beads on cell differentiation, these stimulations also increased the percentages of infected cells, which similarly, was more pronounced at 96 h (Fig 6E and 6F, S8A–S8D Fig). Again, adding of CLI-095 reduced the rates of infection at both 24 h and 96 h (Fig 6E and 6F, S8A–S8C Fig) further confirming the role of TLR4-dependent differentiation in the susceptibility of THP-1 cells to EBOV-infection. Furthermore, the analysis of THP-1 cells stimulated by HPIV3/ΔF-HN/EboGP and GP beads suggests that the observed induction of cell differentiation leading to increased susceptibility to EBOV infection is specifically related to interaction of GP with TLR4. Overall, these results demonstrate that engagement of TLR4 by EBOV GP increases their differentiation, which subsequently leads to their increased susceptibility to the virus.

## Discussion

This study demonstrates, for the first time, that despite the lack of infection of T lymphocytes, EBOV directly binds and induces T cell death. In addition, this study demonstrates that interaction of EBOV GP with TLR4 stimulates differentiation of monocytes, which results in an increased susceptibility to EBOV infection. We show that following EBOV infection, monocytes undergo activation, which is known to lead to secretion of TNFα [49]. TNFα can contribute to activation and bystander death of T lymphocytes, which is consistent with the previously demonstrated effects of TNFα on T cells [50]. Furthermore, we show a direct binding of EBOV to T cells partially involving TLR4 and presumably involving additional ligands, as previous studies have identified the lectins DC-SIGN and L-SIGN [51–53], folate receptor-α [54], Tyro3 receptor tyrosine kinases [55] as attachment factors for EBOV. We demonstrate that the binding triggers the activation of numerous inflammatory signaling pathways including interferon, TLR and cell death signaling pathways as evidenced by the transcriptional profile of EBOV-stimulated T cells. The observed effects of TLR4 inhibitors conclusively demonstrated the role of TLR4 in lymphocyte cell death. Finally, using vectored and bead delivery of GP we demonstrate the direct role of the protein in the induction of both MyD88-independent (TRAM1) and MyD88-dependent activation of the TLR4 signaling pathway and subsequent initiation of cell death pathways.

Previous reports demonstrated the involvement of TNFα in apoptosis [56] as well as in necrosis following accumulation of reactive oxygen species [57]. Necrotic and apoptotic pathways are closely related, and following TLR4 activation, MyD88-dependent signaling pathway has also been reported to trigger necroptosis [58], which also can occur in EBOV-cultured T cell culture as we demonstrated MyD88-dependent activity (Fig 4A). The fact that multiple cell death mechanisms occur in the same EBOV-cultured T cells environment conjugated to the fact that different cell death mechanisms are interconnected between each other [59] could explain the drastic diminution of cell death observed in association with known specific inhibitors of apoptosis, necrosis or TNFα following EBOV-cultured T cells. Of note, HPIV3 also induced some TLR4 signaling (Fig 4A) but only low-level apoptosis (Fig 2) that can be explained by some differences in signaling pathways induced by direct engagement of TLR4 by HPIV3 versus EBOV or by different strength of TLR4 signaling (S4A Fig).

A recent study demonstrated a wide-spread T lymphocyte activation in EBOV-infected patients receiving experimental antibody-based therapies; however, the percentage of CD4^+^ and CD8^+^ T cell responders was limited in comparison to the total percentage of activated T lymphocytes [60]. It was suggested that the activation may result from stimulation with immune complexes formed by the administered EBOV therapeutic monoclonal antibodies or convalescent plasma. However, based on the present data, activation may also be attributed to the activator role of GP.

In parallel with these data, the activation of the TLR4 signaling pathway, sensitivity to TLR4 inhibitors and production of TNFα are routinely associated with LPS-induced bacterial sepsis [61]. Activation of innate immune pathways *via* pattern recognition receptors (PRR) including TLR4 has been shown to lead to systemic inflammation [62]. Previous studies indicated that successfully blocking TLR4 signaling significantly reduces the pathogenesis associated with bacterial sepsis and lethal influenza infection indicating the critical role of this innate immune-signaling pathway in exasperating immunological responses [63, 64]. Our findings suggest that TLR4 inhibitors may be of therapeutic value for the treatment of EBOV patients. For example, our recent study demonstrates a high level of protection against EBOV and the closely related Marburg virus *in vivo* by the TLR4 receptor antagonist Eritoran [65].

Lymphopenia is consistently observed in human and nonhuman primate models following infection with viruses that cause viral hemorrhagic fever (VHF) [4, 66, 67] and therefore, this phenomenon is not restricted to EBOV infection. Furthermore, lymphopenia has been observed during fatalities from several non-VHF-related pathogens or pathologies, including that caused by highly pathogenic influenza virus, West Nile virus and bacterial sepsis [68–70]. Overall, remarkable similarities exist between EBOV-associated symptoms and the pathological features associated with the diseases caused by these non-VHF-related etiological agents including immune suppression, toxic effects due to inflammatory mediators and high viremia in the case of viral infections [66]. As the onset of lymphopenia is highly correlative with fatal outcomes following infection with aforementioned pathogens, identification of factors contributing to T lymphocyte cell death may enable the development of broad acting therapeutics.

Lastly, we note that all current EBOV candidates rely on GP as a sole antigen [71]; with all requiring extremely high doses to achieve protection. For example a recent clinical study utilizing the VSV-vectored EBOV vaccine VSVΔG/ZEBOVGP, which is known to replicate at high levels, required a vaccination dose of 2x10^7^ PFU [72]. The current study demonstrated induction of cell death by VSVΔG/ZEBOVGP (S2A and S2B Fig). It is possible that the high vaccine doses required for the induction of protective immune responses are necessary to compensate for the reduced immunogenicity associated with the immune-modulating effects of GP presented in this study. Furthermore, another clinical trial of this vaccine demonstrated induction of a transient arthritis and dense CD4^+^ T lymphocytic vasculitis suggesting a pathophysiological role of vaccine-induced T lymphocytes. Both effects were related to the EBOV GP component of the vaccine, but not the VSV vector [73]. In addition, HPIV3/ΔF-HN/EboGP, which demonstrated toxic effects associated with GP in this work (Fig 2A and 2B) was also developed as a vaccine candidate [32]. Thus, GP exerts multiple immune modulating effects on immune cells, which may adversely affect the quality of the T cell response although it is also possible that the magnitude of lymphocytes death following vaccinations is modest and does not significantly affect the vaccine efficacy.

The combination of our transcriptome analysis performed on CD4^+^ T cells and the perturbation of signaling cascades associated with cell death following CLI-095 treatment suggest an important role of the GP-TLR4 interaction in the pathogenesis of EBOV infection. Our study highlights diverse strategies used by EBOV to perpetrate lymphopenia through direct and indirect mechanisms, which results in both apoptotic and necrotic T cell death despite the lack of infection. We expanded these studies to include monocytes, which are permissive to EBOV. We found that the increase in activation/differentiation following EBOV-mediated stimulation of TLR4 resulted in a significantly increased rate of infection of monocytes. We note, however, that the effects of TLR-4 on differentiation are likely to be only partial, and other mechanisms involving GP may also contribute it. As mentioned above, several attachment factors have been identified for EBOV; their engagement as well as release of cytokines (e.g. TNFα) are likely to contribute cellular differentiation in an autocrine and/or paracrine manner. These data indicated that infection of monocytes and other cells amplify lymphocytes death by producing more viral particles and also by secreting TNFα and other proteins contributing death of lymphocytes. As TLR4 is expressed by multiple cell types, we suggest that the interactions of GP with TLR4 may have profound effects *in vivo*, and therefore, examination of TLR4 inhibitors as therapeutics for EBOV-infection is warranted. Overall, these data contribute to understanding of the ‘immune paralysis’ during EBOV infections.

## Materials and methods

### Cell lines

Human embryonic kidney 293T (293T), SupT1, THP-1 and Jurkat cell lines were obtained from the American Type Culture Collection. THP-1 MyD88-/- cells were obtained from InVivoGen. 293T were cultured in Dulbecco’s modified Eagle medium (DMEM) supplemented with 10% heat-inactivated fetal bovine serum (HI-FBS) (ThermoFisher Scientific), 1% HEPES (Corning), 1% nonessential amino acids (Sigma-Aldrich), 1% sodium pyruvate (Sigma-Aldrich) and 2% PenStrep mix (ThermoFisher Scientific). THP-1, THP-1 MyD88^-/-^, SupT1 and Jurkat cell lines were cultured in RPMI 1640 (ThermoFisher Scientific) supplemented with 10% HI-FBS and 1% HEPES.

### Viruses, VLPs, EBOV GP beads

Viruses and VLPs used in the study are briefly described in S1 Table. The recombinant EBOV, strain Mayinga, expressing green fluorescent protein (EBOV-GFP) or wild-type (EBOV) were generated as described in our previous study [17]. The recombinant wild type human parainfluenza type 3 (HPIV3) [74], strain JC, was provided by Drs. P. Collins and M. Skiadopoulos (National Institutes of Health). Chimeric HPIV3, in which EBOV GP has been added to the envelope (HPIV3/EboGP) or where the HN and F genes were replaced with that of GP (HPIV3/ΔF-HN/EboGP) were generated in our previous study [32]. All viruses were quantitated by plaque titration in Vero-E6 monolayers as previously described [17]. EBOV VLPs were generated as previously described [75] using mammalian cell codon optimized plasmids expressing EBOV GP pWRG7077:64755-2010-233-1_GP_optVP40 (codop-EBO-GP) and VP40 pWRG7077:64759-2010-233-1-4_VP40_optVP40 provided by Dr. Sina Bavari (U.S. Army Medical Research Institute of Infectious Diseases). His-tagged GP were purchased from Integrated BioTherapeutics) and bound to Dynabeads (ThermoFisher Scientific) following manufacturer’s instructions. Beads were washed with RPMI 1640 supplemented with 10% HI-FBS and 1% HEPES and used at a concentration of 3 beads/cell.

### Analysis of markers of differentiation and EBOV-GFP infection in THP-1 cells

THP-1 cells were plated at 1x10^6^ cells/ml in 24-well plates in medium alone or medium containing CLI-095 (100 ng/ml) for 1 h. Thereafter LPS (500 ng/ml), EBOV (no GFP), EBOV GP beads or HPIV3/ΔF-HN/EboGP were added at MOI 3 PFU/cell and cells were incubated for 24 h or 96 h. To analyze markers of differentiation, cells were harvested, stained with antibodies specific for CD14-BUV395 (BD Biosciences #563561) and CD11b-FITC (BD Biosciences #562793), permeabilized, fixed and stained with antibodies specific for CD68-PE/Cy7 (BD Biosciences #565595). To analyze susceptibility to infection, following stimulation cells were centrifuged for 5 min at 250 g and supernatants were removed, cells were infected with EBOV-GFP at MOI of 3 PFU/cell, and incubated for an additional 48 h. CLI-095 (100 ng/ml) was added 1h prior cells were cultured with conditions. Cells were harvested, fixed and analyzed and analyzed using for markers of differentiation or GFP by FACS Fortessa flow cytometer (BD Biosciences).

### Intracellular staining and secretion of cytokines

SupT1 cells were cultured at 1x10^6^ cell/ml in 24-well plates in medium with or without CLI-095 at 1 μg/ml or anti-TLR4 antibodies at 50 μg/ml for 1 h. Thereafter, cells were mock-treated or treated with TPA (Sigma-Aldrich) (25 ng/ml) / ionomycin (Sigma-Aldrich) (0.5 μM) or EBOV (MOI 3 PFU/cell). Then, cells were treated with Brefeldin-A (Sigma-Aldrich) (10 μg/ml) 1 h post treatment for 24 h. Cells were harvested, stained for intracellular TNFα or IFNγ using anti-TNFα-Pacific Blue (Biolegend #502920) and anti-IFNγ-PE (eBiosciences #12-7319-42) and analyzed by flow cytometry. Supernatants were analyzed for TNFα by Multiplexing LASER Bead Technology by Eve Technologies (Calgary, Canada).

### Isolation and culture of primary T lymphocytes

Buffy coats were obtained from anonymous healthy adult donors according to a clinical protocol approved by the University of Texas Medical Branch at Galveston (UTMB) Institutional Review Board. Peripheral blood mononuclear cells (PBMCs) were isolated by Histopaque (Sigma-Aldrich) gradient as recommended by the manufacturer. CD14^+^ monocytes were isolated from fresh PBMCs, which were subsequently used for isolation of CD3^+^, CD4^+^ and CD8^+^ T lymphocytes by positive selection using magnetic microbeads separation kits (all from Miltenyi). In experiments where only CD4^+^ T cells were isolated, a negative selection CD4^+^ T cells isolation kit (Miltenyi) was used. In some experiments, negative selection of CD3^+^, CD4^+^ or CD8^+^ T cells (Stem Cell Technologies) was used. Purity of the isolated lymphocytes typically ranged from 93 to 95% as determined by flow cytometry.

### Differentiation of monocyte-derived DCs and co-culture with T lymphocytes

CD14^+^ monocytes were cultured in Monocyte-DC Differentiation Medium (Miltenyi) for 7 days to obtain immature DCs. To differentiate immature DCs to mature DCs, Mo-DC Maturation Medium (Miltenyi) was added and cells were incubated for an additional 3 days. Immature and matured DCs were assessed using Mo-DC Differentiation Inspector (Miltenyi) and analyzed by flow cytometry. For co-culture experiments, DCs were combined with autologous cryopreserved CD4^+^ or CD8^+^ T lymphocytes at a 1:1 ratio in 96-well U-bottom plates in RPMI 1640 medium supplemented with 10% HI-FBS. LPS (Invivogen) was added as indicated. T lymphocyte activation was induced with Dynabeads Human Transactivator CD3/CD28 Beads (ThermoFisher Scientific) according to manufacturer’s recommendations.

### Flow cytometry analysis of GP binding

SupT1 cells were plated at the concentration of 1x10^6^ cells per well in U-Bottom 96-well plates (ThermoFisher Scientific) and placed on ice (to prevent internalization of viruses without infection), and EBOV at MOI 1 PFU/cell was added. Cells were incubated for 2 h at 4°C and washed with PBS containing 2% HI-FBS. Thereafter, cells were immunostained with rabbit antibodies raised against EBOV VLP (Integrated BioTherapeutics). After staining, cells were washed three times with PBS containing 2% HI-FBS, fixed in 10% formalin (ThermoFisher Scientific) and stained with goat anti-rabbit antibodies labeled with Alexa-Fluor 647 (ThermoFisher Scientific) and washed again 3 times in PBS with 2% HI-FBS. Flow cytometry was performed using a LSRII Fortessa flow cytometer (BD Biosciences) available at the UTMB Flow Cytometry Core Unit.

### Experiments with inhibitors of cell death and prosurvival cytokines

The inhibitors of necrosis NecroX5 (Enzo Life Sciences), geldanamycin (Invivogen) or N-(3-aminomethyl)benzylacetamindine (Santa Cruz Biotechnology) were used at concentrations 20 μM, 10 μM and 10 μM, respectively. The inhibitor of apoptosis z-VAD-FMK (Affymetrix eBioscience) was used at 20 μM. The inhibitor of TNFα, TNFα antagonist III (Santa Cruz), was used at 1 μM. The TLR4 inhibitor CLI-095 (Invivogen) was used at 100 ng/ml. The inhibitors were added to cell cultures 1 h prior to the addition of EBOV (MOI 1 PFU/cell). The T lymphocyte prosurvival cytokines, IL-4, IL-7 and IL-15 (all R&D Systems) were used at 100 ng/ml, 10 ng/ml, and 100 ng/ml, respectively. In all experiments, inhibitors of cell death and prosurvival cytokines were added 30 minutes prior to the addition of EBOV (MOI 1 PFU/cell).

### Analysis of TLR4 signaling activation and detection of phosphorylated NFκB

Monocytes, THP-1, THP-1 MyD88-/- or SupT1 T lymphocytes were plated at a concentration of 1x10^6^ cells per well in 96- or 24-well plates and mock-treated or treated with CLI-095 at 1 μg/ml for 1h. Then cells were stimulated with HPIV3 WT, HPIV3/EboGP, HPIV3/ΔF-HN/EboGP, EBOV GP beads, EBOV at MOI 0.1, 1 or 3 PFU/cell or mock-stimulated, transfected Poly I:C (10 μg/ml), VLPs at 10 μg/ml or 25 μg/ml, CD3/CD28 beads at a ratio of 1 bead per 3 cells, or LPS at 100 or 500 ng/ml and harvested at the indicated time points. Cell were collected at the indicated time points and lysed in RIPA buffer (ThermoFisher Scientific). Proteins were separated by SDS-PAGE using gradient 4–12% gels (ThermoFisher Scientific) and transferred to nitrocellulose membranes (ThermoFisher Scientific) using the I-blot system (ThermoFisher Scientific). Membranes were blocked with 5% milk and 0.1% Tween-20 in PBS for 1 h at 37°C and stained with antibodies specific for the following molecules: TRAM1 (Abcam #ab96106), phosphorylated TRAM1 (FabGennix #PTRAM-140AP), MyD88 (#4283S), IRAK4 (#4363S), phosphorylated IRAK4 (#11927S), Pyk2 (#3090S), phosphorylated Pyk2 (#3291S), p38 (#8690S), phosphorylated p38 (#4511S), NFκB (#6956S), phosphorylated NFκB (#3033S) and GAPDH (#8884S) (all Cell Signaling Technology) diluted according manufacturer’s recommendations in PBS with 0.1% of Tween-20.

### Cell death analysis

Primary T lymphocytes, lymphoid cell lines or PBMC were stained with CFSE (ThermoFisher Scientific) to monitor cell proliferation as recommended by manufacturer. The activity of caspase-8 and caspase-9 was determined at the indicated time points by flow cytometry using Vybrant FAM Caspase-8 kit (ThermoFisher Scientific) or CaspGLOW Active Caspase-8 Staining kit (eBioscience) and Red FLICA Caspase-9 Assay kit (Immunochemistry Technologies). Thereafter, cells were stained with a combination of the following antibodies: anti-CD3 clone UCHT1, labeled with BUV395, anti-CD3 clone UCHT1, labeled with Pacific Blue, anti-CD4 clone OKT4, labeled with PerCP-Cy5.5, anti-CD8 clone RPA-T8, labeled with PerCP-Cy5.5, anti-CD8 clone RPA-T8, labeled with APC, and also with Annexin-V labeled with PE (all BD Biosciences). As the study was done under BSL-4 biocontainment, and due to biosafety regulations, cells analyzed by flow cytometry must be fixed with paraformaldehyde. Since apoptotic cells are positive for Annexin V, co-staining of cells with Annexin V and Live/Dead stain instead of PI provides the opportunity to discern apoptotic from necrotic cells [76]. As both PI and 7-AAD are poorly compatible with fixation protocols, Live/Dead staining which is compatible with formalin fixation and similarly to PI and 7AAD stains dead cells, was used. Following surface receptor staining, Live/Dead (ThermoFisher Scientific) staining was performed according manufacturer’s recommendation and cells were fixed with 10% formalin. Flow cytometery was performed using a FACS Fortessa instrument (BD Biosciences). Jurkat T cells were plated at 1x10^6^ cells per well of a 24-well plate and HPIV3 or HPIV3/ΔF-HN/EboGP was added at a MOI 1 PFU/cell. Cells were incubated for 7 days at 37°C. As a positive control for activation of caspase-3, -8 and -9, cells were treated with staurosporine (Sigma-Aldrich) at 1 μM for 6 h. After 7 days, cells were harvested, washed three times with PBS and lysed in RIPA lysis buffer supplemented with 4x Laemmli buffer (ThermoFisher Scientific). Western blot analysis was performed using anti-caspase-3 (Santa Cruz Biotechnology sc-271028), anti-caspase-8 (Santa Cruz Biotechnology sc-81657), and anti-caspase-9 (Cell signaling #9502) antibodies. Densitometric analyses of active caspase-3, active caspase-8, active caspase-9 were performed using ImageJ software (NIH) and normalized using GAPDH. SupT1 cells were infected with VSVΔG/ZEBOVGP at 1 or 3 PFU/cell, incubated for 4 days at 37°C, stained with Live/Dead and analyzed by flow cytometry as described above.

### Analysis of EBOV GP-TLR4 binding by immunoprecipitation

TLR4 expression in purified T lymphocyte populations and cell lines was determined by western blot analysis using anti-TLR4 antibody (Santa Cruz Biotechnology, #sc-293072). GP-TLR4 binding was determined by co-transfecting 293T cells with mammalian codon-optimized plasmids encoding EBOV GP or VP40, as well as the plasmids expressing TLR4 (Addgene, #20863) or TLR4 FLAG-tagged protein (TLR4-FLAG) (Addgene, #42646) using TransIT-LT1 reagent (Mirus) for 48 h at 37°C. Cells were lysed with 500 μl of RIPA lysis buffer supplemented with Protease and Phosphatase Inhibitor Cocktail (ThermoScientific) for 30 min at 4°C. Lysates were centrifuged at 400 x g at 4°C for 10 minutes. Then, 50 μl of cleared supernatants were kept for protein expression analysis while the remaining 450 μl were utilized for immunoprecipitation assays. Supernatants were incubated with monoclonal antibodies specific for TLR4 (Santa Cruz Biotechnology, #sc-293072) or FLAG, clone M2 (Sigma-Aldrich) and incubated for 2 h at 4°C with rotation, followed by addition of protein G agarose beads (ThermoFisher Scientific) and overnight incubation at 4°C on a rotating platform. Beads were centrifuged at 2,500 x g at 4°C, washed with RIPA buffer (ThermoFisher Scientific) three times, and resuspended in 50 μl of Laemmli lysis buffer (ThermoFisher Scientific) for western blot analysis. The following antibodies (all Integrated BioTherapeutics) were used for western blot analysis: rabbit anti-GP (#0301–015), rabbit anti-VP40 (#0301–010) and rabbit anti-EBOV VLP (#01–0004). HRP-conjugated Secondary (Santa Cruz Biotechnology) antibodies were used to visualize bands following the addition of ECL reagent (ThermoFisher Scientific).

### Confocal microscopy

293T cells transfected with a TLR4-expressing plasmid (as described above), THP-1, SupT1, Jurkat and primary CD4^+^ T cells were plated at 1x10^5^ cells/ml and HPIV3/ΔF-HN/EboGP and EBOV or EBOV GP beads were added at MOI 5 PFU/cell or 3 beads/cell respectively. Cells were incubated for 2 h on ice or 2 h at 37°C, washed with PBS with 2% HI-FBS, fixed with 10% formalin and loaded on positively charged slides (ThermoFisher Scientific) and dried overnight. Following rehydratation, cells were permeabilized with PBS 0.5% Triton X100 (Alfa Aesar) for 15 minutes. Cells were then washed with PBS and incubated with 0.5 M glycine in PBS for 30 minutes at room temperature before performing antigen blocking using 5% donkey serum diluted PBS with 1% BSA and 0.1% Triton X100 (PBS-BSA-TX100) for 1 h. Anti-EBOV VLP serum (Integrated BioTherapeutics) was diluted at 1:100, anti-LAMP1 and anti-Rab7 were diluted at 1:12.5 and anti-TLR4 was diluted at 1:50 using PBS-BSA-TX100 and put on the slides for 1 h. Then, slides were washed with PBS with 0.1% Triton X100 and incubated with secondary donkey anti-rabbit antibodies conjugated with AlexaFluor 647 (ThermoFisher Scientific) diluted at 1:200 in PBS-BSA-TX100 for an additional 1 h before being washed as above. Next, cells were incubated with 6-diamin-2-phenylindole-dihydrochloride (DAPI) (ThermoFisher Scientific) at 1 μg/ml for 2 minutes and washed with PBS. The coverslips were mounted onto microscope slides using PermaFluor mounting medium (ThermoFisher Scientific). Laser scanning confocal microscopy was performed on Olympus FV1000 confocal microscope housed in the Galveston National Laboratory. Laser beams with 405 nm wavelengths were used for DAPI excitation, and 635 nm for AlexaFluor 647 excitation. Emission filters were 425/25 nm for DAPI and 610/50 nm for AlexaFluor 647 detection, respectively. All images were acquired using a 60x oil objective.

### Transcriptome deep sequencing

Isolated CD4^+^ T cells from four donors were cultured in RPMI1640 medium in the presence or absence of EBOV at MOI 3 or LPS at 500 ng/ml. 24 and 96 h post stimulation, cells were washed with PBS, lysed in 1 ml of TRIzol (ThermoFisher Scientific) and stored at -80°C. RNA isolation was performed using Direct-zol RNA MiniPrep kit (Zymo Research). mRNA libraries were constructed following analysis with an Agilent 2100 Bioanalyzer (nanochip format). Libraries were constructed using the Kapa Stranded mRNA-Seq Kit (Kapa Biosystems) according to the manufacturer’s instructions before being quality controlled and quantitated using the BioAnalzyer 2100 system and QuBit (Invitrogen). The libraries were clonally amplified and sequenced on an Illumina NextSeq 500 to achieve a target density of approximately 200K-220K clusters/mm ^2^ on the flow cell with dual indexed paired end sequencing at a 75 bp length using NextSeq 500 NCS v1.3 software. Raw reads (75 bp) had their adapter sequences removed.

### Read processing

FastQC (http://www.bioinformatics.babraham.ac.uk/projects/fastqc/) was used for general quality control of the raw reads. Bowtie (v2.1.0) was utilized to remove ribosomal RNA using an index of human, mouse, and rat rRNA sequences [77]. Reads were then mapped against a human reference genome (hg19, build GRCh37, from the UCSC genome browser (http://genome.ucsc.edu) using STAR (v2.4.0h1) [78]. From this alignment, quantitative gene counts were produced using HTSeq (http://www-huber.embl.de/users/anders/HTSeq/doc/overview.html) utilizing the human annotation associated with the genome [79].

### Differential expression analysis

Gene counts for 25,237 genes for each sample were loaded into R (http://www.r-project.org/). Counts across samples were normalized with edgeR (version 3.10.2) using the weighted trimmed mean of M-values while genes with no counts or at least three sample with counts were removed [80] leaving 14,932 genes with an average of ~10^6^ reads per sample. Differentially expressed genes were identified using edgeR between treatments and time points and defined by using an absolute fold change cutoff of 1.5 and a p-value of ≤ 0.05 after adjustment using the Benjamini-Hochberg multiple testing correction. Additional clustering, creation of heatmaps, and other statistical analyses were performed using R.

### Functional enrichment analysis

Functional analysis of the differential gene expression data was performed with QIAGEN’s Ingenuity Pathway Analysis (IPA, QIAGEN Redwood City, www.qiagen.com/ingenuity). Functional annotation of genes for specific biological functions was assigned through querying AmiGO (version 2.20) [81]. Gene names were collected by searching for human genes with search queries “cell death”, “necrosis”, “apoptosis”, and “TLR”. Functional enrichment analysis using GO terms was performed using the PANTHER Overrepresentation Test (release 20150430) from the GO Ontology database released 2015-08-06 against a reference of all *Homo sapiens* genes using the complete GO biological processes annotation set [82].

### Network analysis

Molecules from both the experimental expression data and the IPA Knowledge base were added to the networks that were then assigned a score derived from its p-value. IPA network scores of 2 or higher have at least a 99% confidence interval of not being generated by chance alone. Genes contributing to the construction of the TLR4 and necrosis networks were created from stricter absolute log_2_ fold change cutoffs of > 2 fold to focus on the interactions between the most highly activated genes. Molecular activity prediction (MAP) analysis was also performed on the cell death network. MAP analysis uses the IPA Knowledge Base to predict the upstream and downstream effects of activating or inhibiting a molecule in the network.

### Statistical analyses

Each independent experiment was performed in triplicate to rule out experimental bias or random error. Statistical methods used were described in Figure Legends using GraphPad Prism 6 (GraphPad Software). *P* values of <0.05 were considered statistically significant. Mean and standard error of the mean (SE) were calculated for all graphs.

### BSL-4 work

All work with EBOV was performed within the Galveston National Laboratory biosafety level 4 laboratories. All staff had the appropriate training and U.S. government permissions and registrations for work with EBOV.

## Supporting information

S1 Table Viruses and virus-like particles used in the study. (DOCX)

S1 Fig EBOV-induced cell death and proliferation. **A**. Representative examples of flow cytometry analysis of CD4^+^ T lymphocytes cultured with EBOV at 3 PFU/cell for cell death and proliferation, percentages of gated cells indicated. **B**. Representative examples of flow cytometry analysis of CD4^+^ T lymphocytes cultured with EBOV at MOI 1 or 3 PFU/cell for cell death and proliferation, percentages of gated cells are indicated. **C.** Percentages of annexin V^+^ CD8^+^ T lymphocytes following culture with EBOV-infected or mock-infected immature or mature DCs or CD3/CD28 beads determined by flow cytometry. **D.** Percentages of proliferated CD8^+^ T lymphocytes cultured alone or with EBOV-infected DCs determined by flow cytometry. **E**, **F**. Effects of prosurvival mediators on CD4^+^ (E) and CD8^+^ (F) T lymphocytes exposed to EBOV: ratios of EBOV-exposed annexin V^+^ cells cultured in the presence of prosurvival cytokines, immature DCs (imDCs) or mature DCs (mDCs) to similarly treated cells not exposed to EBOV determined by flow cytometry. C-F, Mean values based on triplicate samples ±SE with *P* values: * P<0.05, ** P<0.01, *** P<0.001, **** P<0.0001, Student T-Test for comparisons to incubations without EBOV (C, D) or without the indicated treatments (E, F). Representative data from one of two independent experiments.
(TIF)

S2 Fig EBOV GP induces T lymphocyte cell death. **A, B**. Analysis of dead SupT1 cells following a 4 day-long incubation with VSVΔG/ZEBOVGP at low and high doses (MOI of 1 and 3 PFU/cell, respectively): representative primary data (A) and mean values ±SE (B) based on triplicate samples, representative data from one of two independent experiments. *** P<0.001, **** P<0.0001 (Student T-test).
(TIF)

S3 Fig Activation of NFkB and induction of cell death via TLR4 signaling. **A**. Western blot analysis of GP biding to His-beads. **B**. Western blot analysis of p-p65 at the indicated time points after stimulation with LPS or EBOV VLPs. Representative data from one of two independent experiments. **C.** Flow cytometry analysis of CD4^+^ T lymphocytes cultured with EBOV or LPS in the presence or absence of CLI-095 for 4 days. The percentages of dead cells, cells positive for active caspase-8 or caspase-9 and proliferated cells are indicated. A-C, representative primary data from one of two independent experiments.
(TIF)

S4 Fig Transcriptome analysis of T cells exposed to EBOV. **A.** Heatmap showing fold change differences between EBOV-infected and LPS-stimutlated versus mock-treated samples on days 1 and 4 for 265 genes that are differentially expressed between EBOV- and mock-infected samples at 24 h using a 1.5-fold change cutoff and an adjusted p-value of 0.05. Genes were chosen based on a search for specific biological functions: apoptosis, necrosis, TLR, and cell death within the AmiGO2 Gene Ontology database. Heatmap shows log_2_ fold changes of genes relative to the average baseline with red representing up-regulation and blue representing down-regulation. **B.** Networks of interactions related to apoptosis (purple), necrosis (yellow) and TLR4 (grey) pathways built from differentially expressed genes from EBOV-infected to mock samples at 24 h. Solid lines represent direct interactions and dotted lines represent indirect interactions from IPA’s Knowledge Base. Expression data from EBOV-infected samples relative to mock samples at 24 h is overlaid onto each gene where red represents relative up-regulation and blue represents relative down-regulation. **C**. Pathways triggered by EBOV-stimulation. Top twenty significantly enriched pathways induced following EBOV-stimulation of isolated CD4^+^ T cells. P-values represent the likelihood the association of these genes and pathways are due to chance (a p-value of 0.01 has a negative log p-value of 2). Ratios, shown by the dots, are calculated by the number of genes in each group belonging to the pathway over the total number of genes comprising that pathway. Predicted up-regulation of each pathway is represented by orange coloration, with darker colors representing increased up-regulation. The bars are the p-value (on top) and the dots are the ratio (on bottom).
(TIF)

S5 Fig Anti-TLR4 antibodies reduce cytokine production. Flow cytometry analysis of the percentages of TNFα^+^ (left panel) and IFNγ^+^ (right panel) SupT1 cells cultured with medium (mock), TPA/ionomycin or EBOV with or without anti-TLR4 antibodies for 24 h: representative primary data (**A**) and mean values ±SE based on triplicate samples (**B**). * P<0.05, ** P<0.01, *** P<0.001, ns, non-significant (Student T-Test). One of two independent experiment is shown. Representative data from one of two independent experiments.
(TIF)

S6 Fig EBOV promotes differentiation and infection of THP-1 cells through TLR4 activation. **A.** Schematic representation of the experimental design to evaluate activation of THP-1 cells by LPS, EBOV or HPIV3/ΔF-HN/EboGP in the presence or absence of CLI-095. **B.** Representative flow cytometry data on analysis of THP-1 cells treated or mock-treated with CLI-095 and cultured with LPS, EBOV or HPIV3/ΔF-HN/EboGP for 24 h or 96 h. **C.** Percentages of CD68^+^ THP-1 cells after treatment with LPS, EBOV or HPIV3/ΔF-HN/EboGP with or without CLI-095 for 24 h or 96 h. Black asterisks indicate differences between CLI-095 treated and untreated cells, red asterisks indicate differences between CLI-095 untreated stimulated and mock-stimulated cells. **D**–**G**, Quantitative data showing the percentages of CD14^+^ (D), CD11b^+^ (E), CD68^+^ (F) or EBOV-GFP^+^ (G) THP-1 cells following incubation with LPS, EBOV or HPIV3/ΔF-HN/EboGP for 24 h or 96 h in the absence of CLI-095. C-G: ** P<0.01, *** P<0.001, **** P<0.0001. Asterisks indicate differences between 24 and 96 h. Mean values based triplicate samples ±SE with P values (Two-Way ANOVA followed by a Tukey’s Multiple Comparisons Test and multiple T-tests). C-G, Representative data from 2 independent experiments.
(TIF)

S7 Fig EBOV GP promotes differentiation of THP-1 cells through TLR4 activation. THP-1 cells were treated or mock-treated with CLI-095, cultured with EBOV GP beads or empty beads for 24 h (**A-D**) or 96 h (**E-H)** and analyzed by flow cytometry for the indicated markers. Representative flow cytometry data (A, E) with the percentages of the gated populations indicated, and quantitative data showing comparisons of CLI-095-treated and untreated cells (B, C, D, F, G, H), and treatments for 24 h versus 96 h without CLI-095 treatment (**I, J, K**). Black asterisks, difference to CLI-095-treated cells, red asterisks, difference to mock-stimulated cells. Mean values based on triplicate samples ±SE. * P<0.05, ** P<0.01, *** P<0.001, **** P<0.0001, ns, non-significant. Two-Way ANOVA followed by a Tukey’s Multiple Comparisons Test and multiple T-tests. B-D, F-K, Representative data from two independent experiments.
(TIF)

S8 Fig EBOV GP promotes infection of THP-1 cells through TLR4 activation. Effects of GP beads on EBOV-GFP infection in THP-1 cells pretreated with CLI-095 analyzed by flow cytometry at 24 and 96 h post infection. **A**. representative flow cytometry data with the percentages of gated populations indicated. **B**–**D**. Percentages of GFP^+^ cells at 24 h (B), 96 h (C) and comparison of CLI-095-treated samples only and 24 h and 96 h (D). B, C, black asterisks, differences between CLI-095-treated and untreated cells; red asterisks, difference to mock-infected cells. D, asterisks, difference between 24 h and 96 h infection. B-D, Mean values based on three samples per group ±SE with *P* values calculated using Two-Way ANOVA followed by a Tukey’s Multiple Comparisons Test and multiple T-tests. * P<0.05, ** P<0.01, *** P<0.001. B-D, Representative data from two independent experiments.
(TIF)
