# Supplementary material for: Ebola virus glycoprotein directly triggers T lymphocyte death despite of the lack of infection
Source: PLoS Pathog. 2017 May 22;13(5):e1006397. doi: 10.1371/journal.ppat.1006397 (PMC5456411; doi:10.1371/journal.ppat.1006397)
Supplement: S1 Table — (DOCX) [file ppat.1006397.s001.docx]

**S1 Table.** Viruses and virus-like particles used in the study.

| **Virus or virus-like particle abbreviation** | **Virus or virus-like particle description** | **Reference** |
| --- | --- | --- |
| HPIV3 | Human parainfluenza virus type 3 strain JC | [1] |
| HPIV3/EboGP | HPIV3-vectored EBOV vaccine construct expressing EBOV GP | [2] |
| HPIV3/ΔF-HN/EboGP | HPIV3-vectored EBOV vaccine construct which represents a chimeric virus lacking the F and HN envelope proteins of HPIV3 and carrying EBOV GP as the only envelope protein | [3] |
| EBOV | Recombinant EBOV strain Mayinga | [4] |
| EBOV-GFP | Recombinant EBOV strain Mayinga expressing GFP | [5] |
| VSV/∆G/ZEBOVGP | VSV-vectored EBOV vaccine construct which represents a chimeric VSV lacking the G envelope protein of VSV and carrying EBOV GP as the only envelope protein. | [6] |
| VLP | Virus-like particles which include EBOV GP, VP40 and NP. | [7] |
