# Supplementary figures and images for: Ebola virus glycoprotein directly triggers T lymphocyte death despite of the lack of infection
Source: PLoS Pathog. 2017 May 22;13(5):e1006397. doi: 10.1371/journal.ppat.1006397 (PMC5456411; doi:10.1371/journal.ppat.1006397)

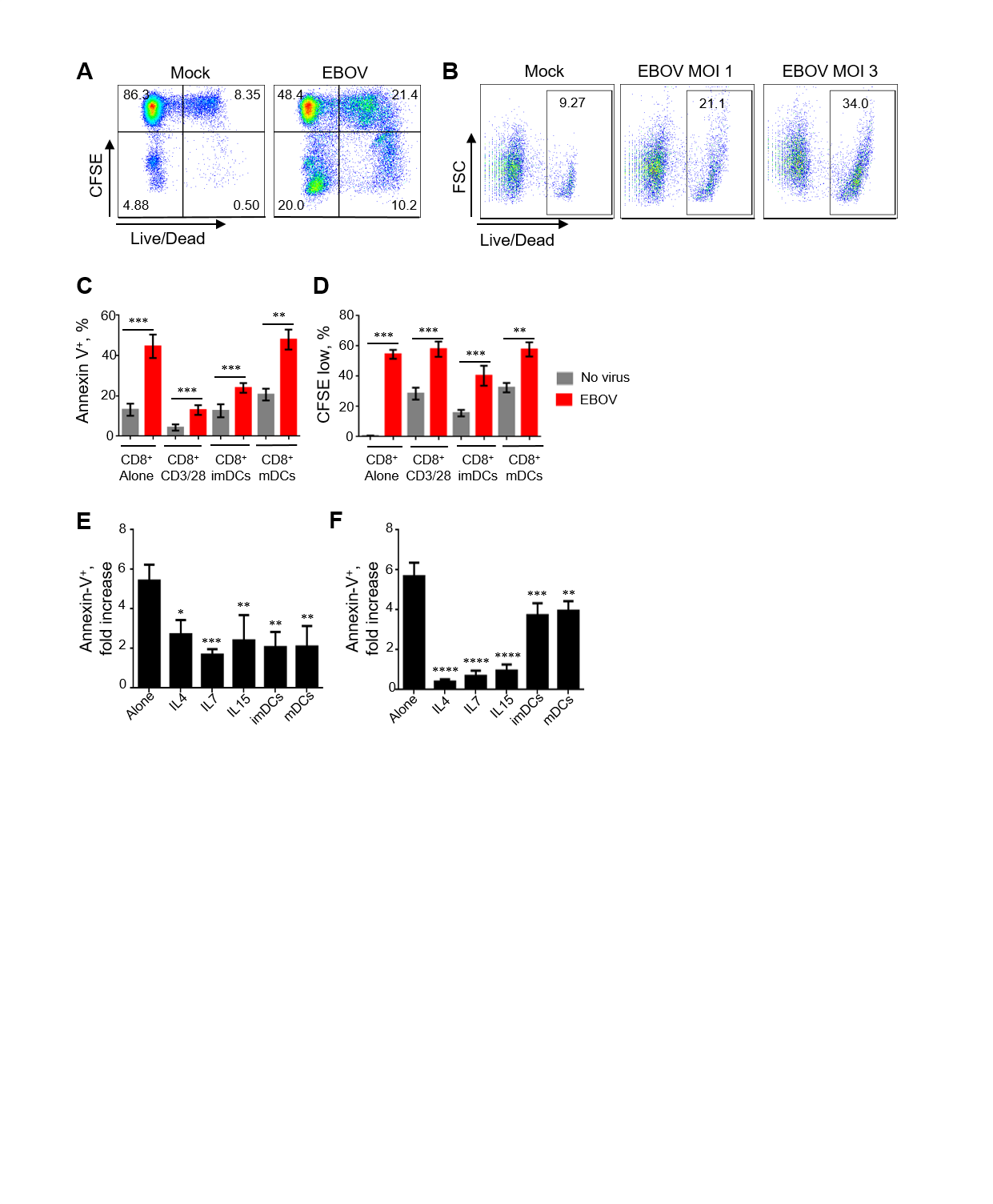

Supplement: S1 Fig — A. Representative examples of flow cytometry analysis of CD4+ T lymphocytes cultured with EBOV at 3 PFU/cell for cell death and proliferation, percentages of gated cells indicated. B. Representative examples of flow cytometry analysis of CD4+ T lymphocytes cultured with EBOV at MOI 1 or 3 PFU/cell for cell death and proliferation, percentages of gated cells are indicated. C. Percentages of annexin V+ CD8+ T lymphocytes following culture with EBOV-infected or mock-infected immature or mature DCs or CD3/CD28 beads determined by flow cytometry. D. Percentages of proliferated CD8+ T lymphocytes cultured alone or with EBOV-infected DCs determined by flow cytometry. E, F. Effects of prosurvival mediators on CD4+ (E) and CD8+ (F) T lymphocytes exposed to EBOV: ratios of EBOV-exposed annexin V+ cells cultured in the presence of prosurvival cytokines, immature DCs (imDCs) or mature DCs (mDCs) to similarly treated cells not exposed to EBOV determined by flow cytometry. C-F, Mean values based on triplicate samples ±SE with P values: * P<0.05, ** P<0.01, *** P<0.001, **** P<0.0001, Student T-Test for comparisons to incubations without EBOV (C, D) or without the indicated treatments (E, F). Representative data from one of two independent experiments. (TIF) [file ppat.1006397.s002.tif]

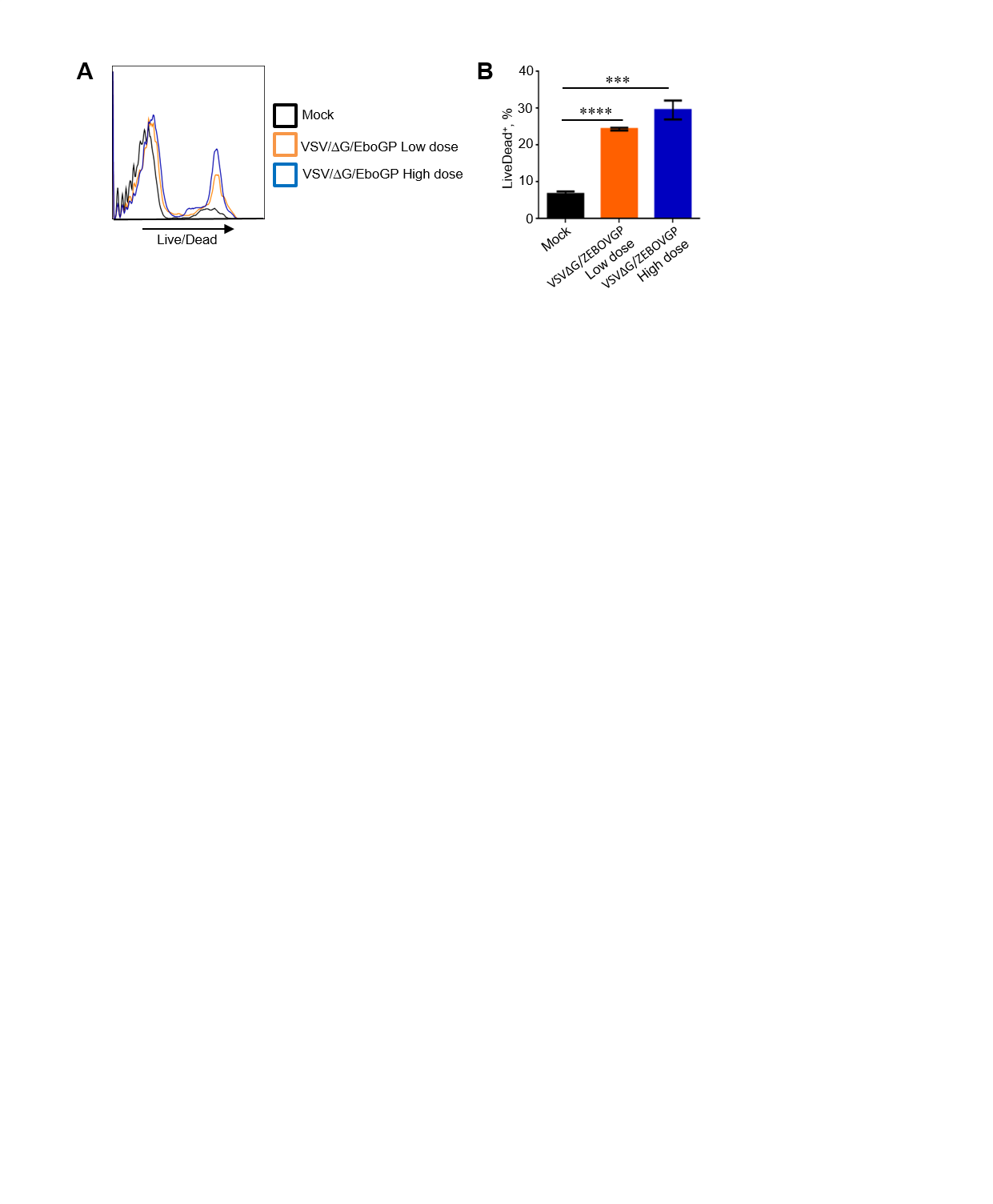

Supplement: S2 Fig — A, B. Analysis of dead SupT1 cells following a 4 day-long incubation with VSVΔG/ZEBOVGP at low and high doses (MOI of 1 and 3 PFU/cell, respectively): representative primary data (A) and mean values ±SE (B) based on triplicate samples, representative data from one of two independent experiments. *** P<0.001, **** P<0.0001 (Student T-test). (TIF) [file ppat.1006397.s003.tif]

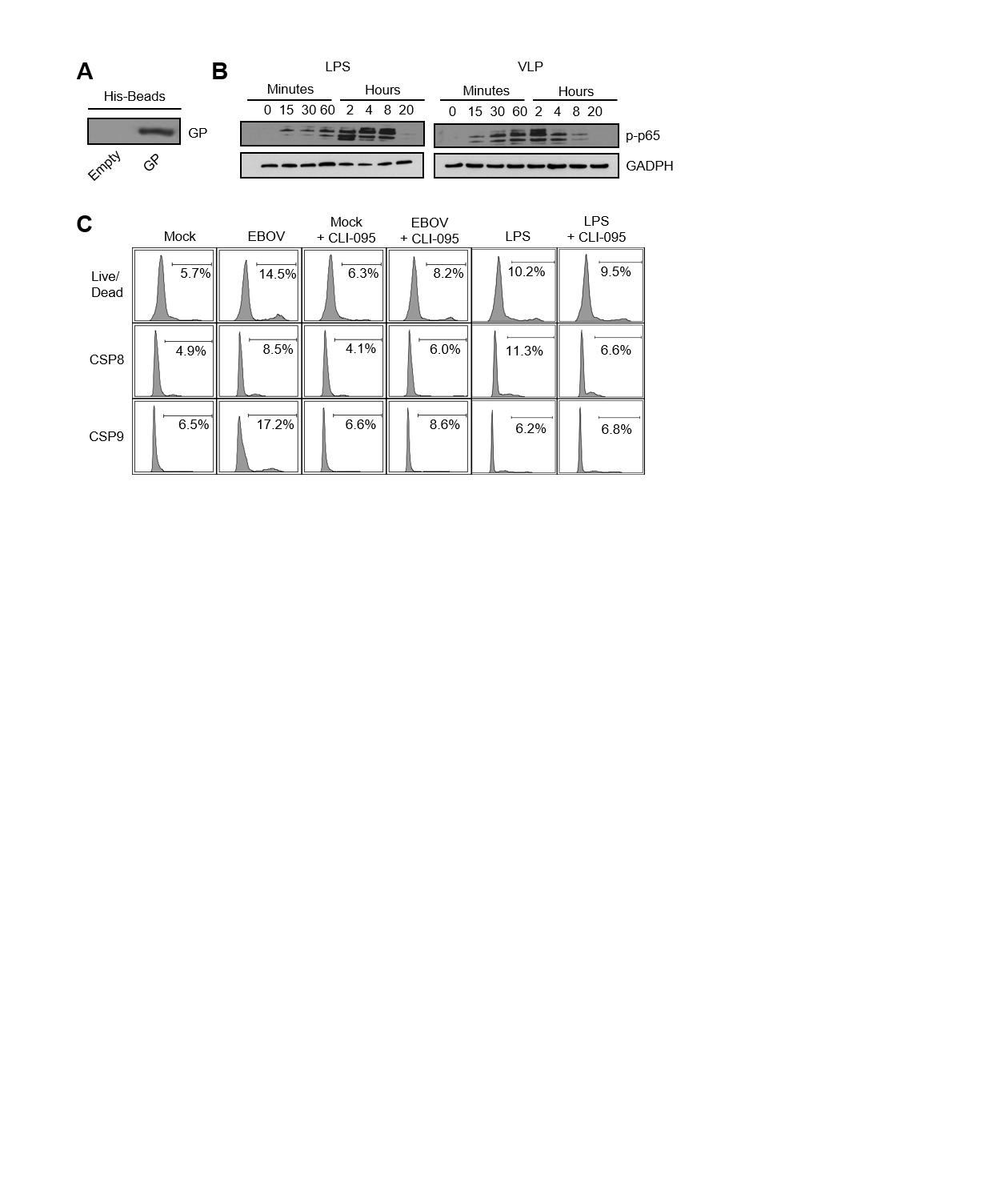

Supplement: S3 Fig — A. Western blot analysis of GP biding to His-beads. B. Western blot analysis of p-p65 at the indicated time points after stimulation with LPS or EBOV VLPs. Representative data from one of two independent experiments. C. Flow cytometry analysis of CD4+ T lymphocytes cultured with EBOV or LPS in the presence or absence of CLI-095 for 4 days. The percentages of dead cells, cells positive for active caspase-8 or caspase-9 and proliferated cells are indicated. A-C, representative primary data from one of two independent experiments. (TIF) [file ppat.1006397.s004.tif]

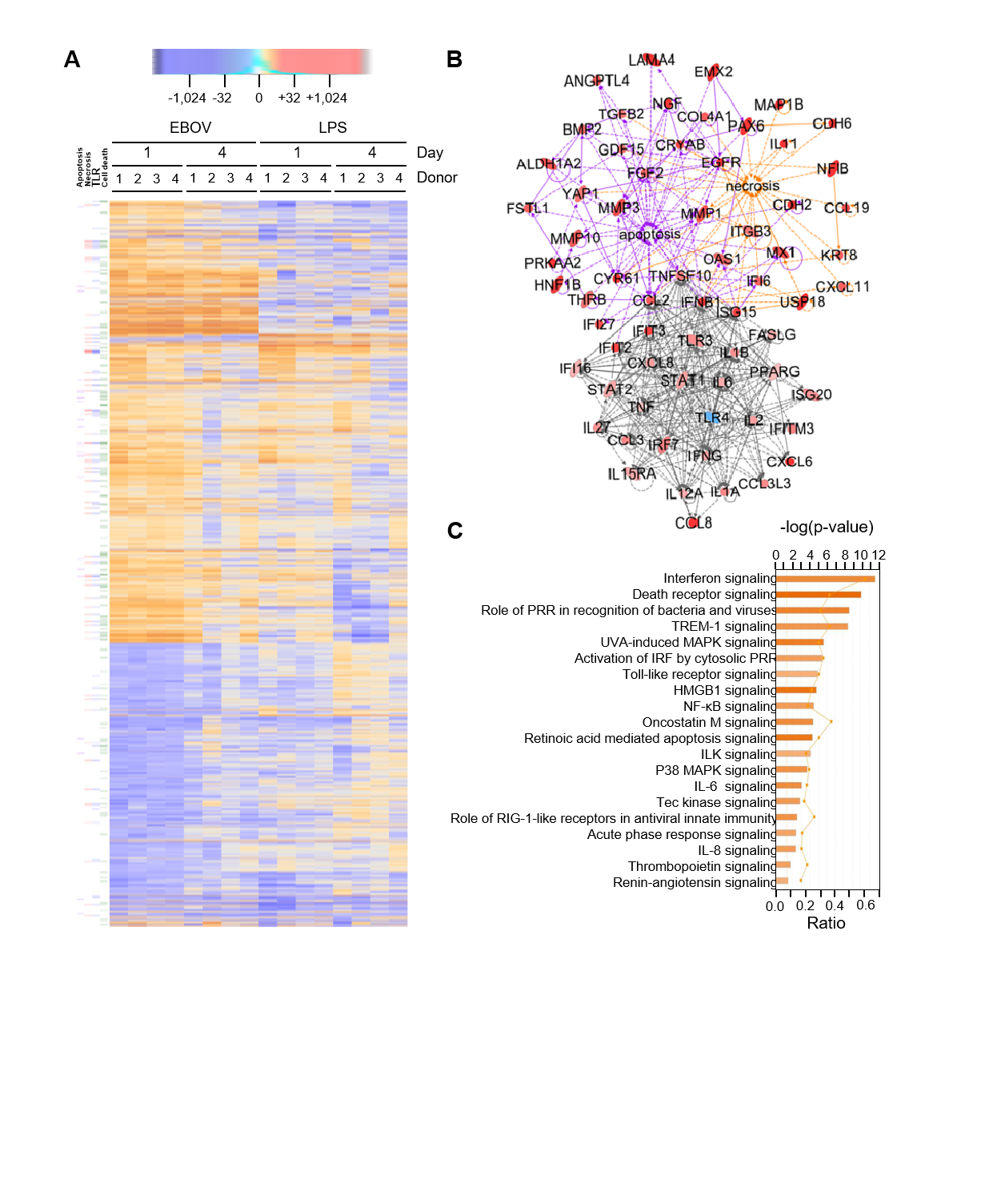

Supplement: S4 Fig — A. Heatmap showing fold change differences between EBOV-infected and LPS-stimutlated versus mock-treated samples on days 1 and 4 for 265 genes that are differentially expressed between EBOV- and mock-infected samples at 24 h using a 1.5-fold change cutoff and an adjusted p-value of 0.05. Genes were chosen based on a search for specific biological functions: apoptosis, necrosis, TLR, and cell death within the AmiGO2 Gene Ontology database. Heatmap shows log2 fold changes of genes relative to the average baseline with red representing up-regulation and blue representing down-regulation. B. Networks of interactions related to apoptosis (purple), necrosis (yellow) and TLR4 (grey) pathways built from differentially expressed genes from EBOV-infected to mock samples at 24 h. Solid lines represent direct interactions and dotted lines represent indirect interactions from IPA’s Knowledge Base. Expression data from EBOV-infected samples relative to mock samples at 24 h is overlaid onto each gene where red represents relative up-regulation and blue represents relative down-regulation. C. Pathways triggered by EBOV-stimulation. Top twenty significantly enriched pathways induced following EBOV-stimulation of isolated CD4+ T cells. P-values represent the likelihood the association of these genes and pathways are due to chance (a p-value of 0.01 has a negative log p-value of 2). Ratios, shown by the dots, are calculated by the number of genes in each group belonging to the pathway over the total number of genes comprising that pathway. Predicted up-regulation of each pathway is represented by orange coloration, with darker colors representing increased up-regulation. The bars are the p-value (on top) and the dots are the ratio (on bottom). (TIF) [file ppat.1006397.s005.tif]

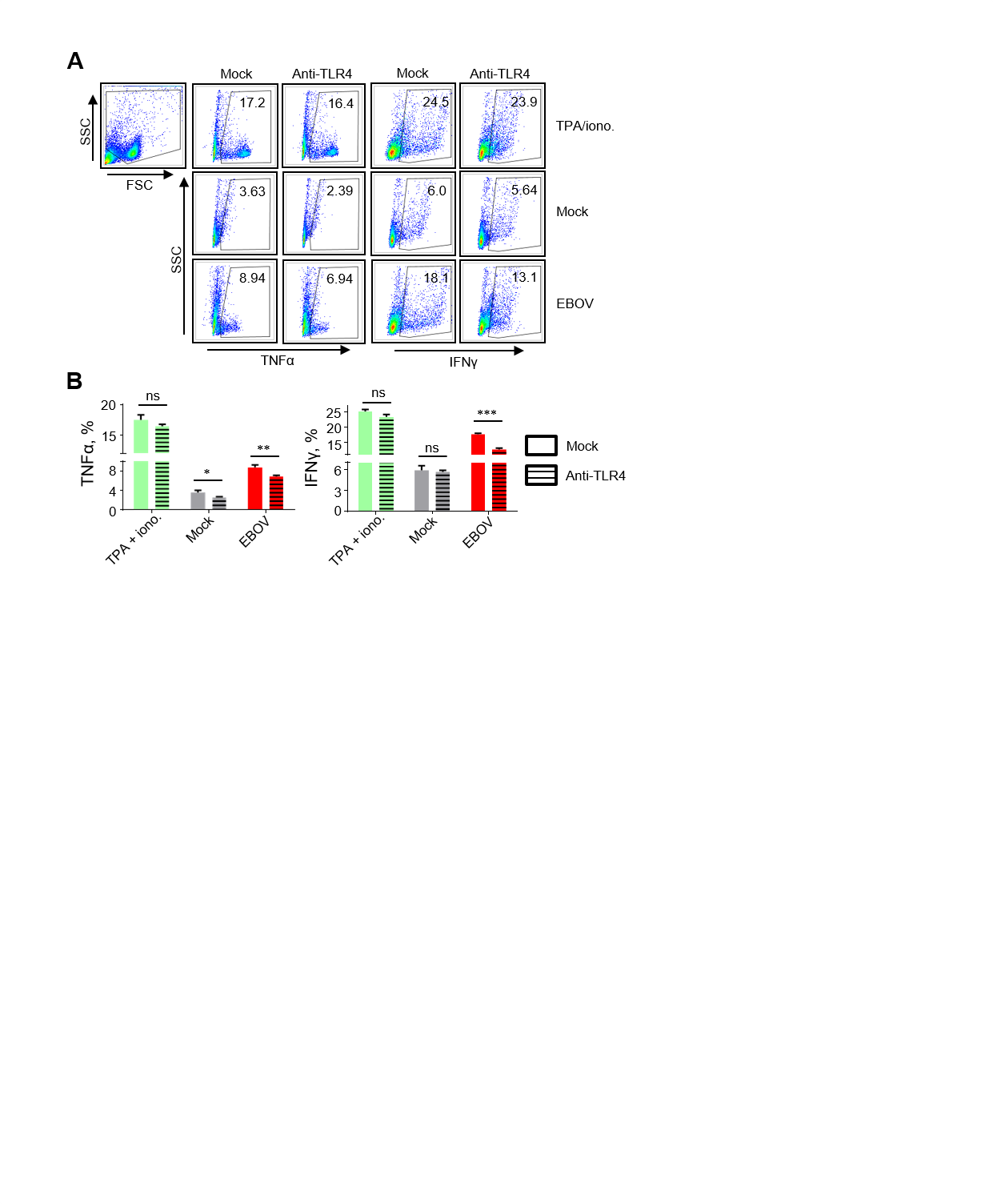

Supplement: S5 Fig — Flow cytometry analysis of the percentages of TNFα+ (left panel) and IFNγ+ (right panel) SupT1 cells cultured with medium (mock), TPA/ionomycin or EBOV with or without anti-TLR4 antibodies for 24 h: representative primary data (A) and mean values ±SE based on triplicate samples (B). * P<0.05, ** P<0.01, *** P<0.001, ns, non-significant (Student T-Test). One of two independent experiment is shown. Representative data from one of two independent experiments. (TIF) [file ppat.1006397.s006.tif]

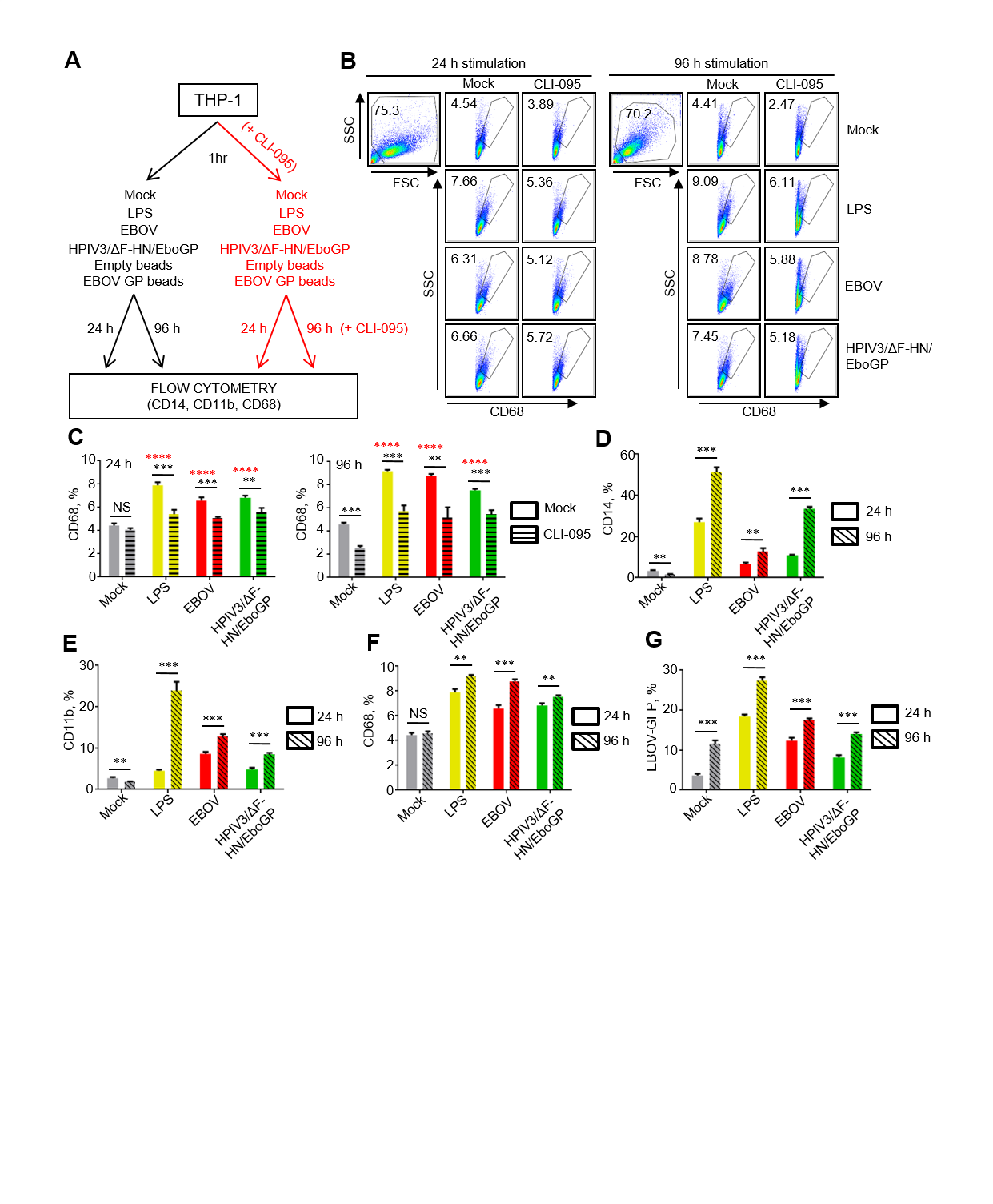

Supplement: S6 Fig — A. Schematic representation of the experimental design to evaluate activation of THP-1 cells by LPS, EBOV or HPIV3/ΔF-HN/EboGP in the presence or absence of CLI-095. B. Representative flow cytometry data on analysis of THP-1 cells treated or mock-treated with CLI-095 and cultured with LPS, EBOV or HPIV3/ΔF-HN/EboGP for 24 h or 96 h. C. Percentages of CD68+ THP-1 cells after treatment with LPS, EBOV or HPIV3/ΔF-HN/EboGP with or without CLI-095 for 24 h or 96 h. Black asterisks indicate differences between CLI-095 treated and untreated cells, red asterisks indicate differences between CLI-095 untreated stimulated and mock-stimulated cells. D–G, Quantitative data showing the percentages of CD14+ (D), CD11b+ (E), CD68+ (F) or EBOV-GFP+ (G) THP-1 cells following incubation with LPS, EBOV or HPIV3/ΔF-HN/EboGP for 24 h or 96 h in the absence of CLI-095. C-G: ** P<0.01, *** P<0.001, **** P<0.0001. Asterisks indicate differences between 24 and 96 h. Mean values based triplicate samples ±SE with P values (Two-Way ANOVA followed by a Tukey’s Multiple Comparisons Test and multiple T-tests). C-G, Representative data from 2 independent experiments. (TIF) [file ppat.1006397.s007.tif]

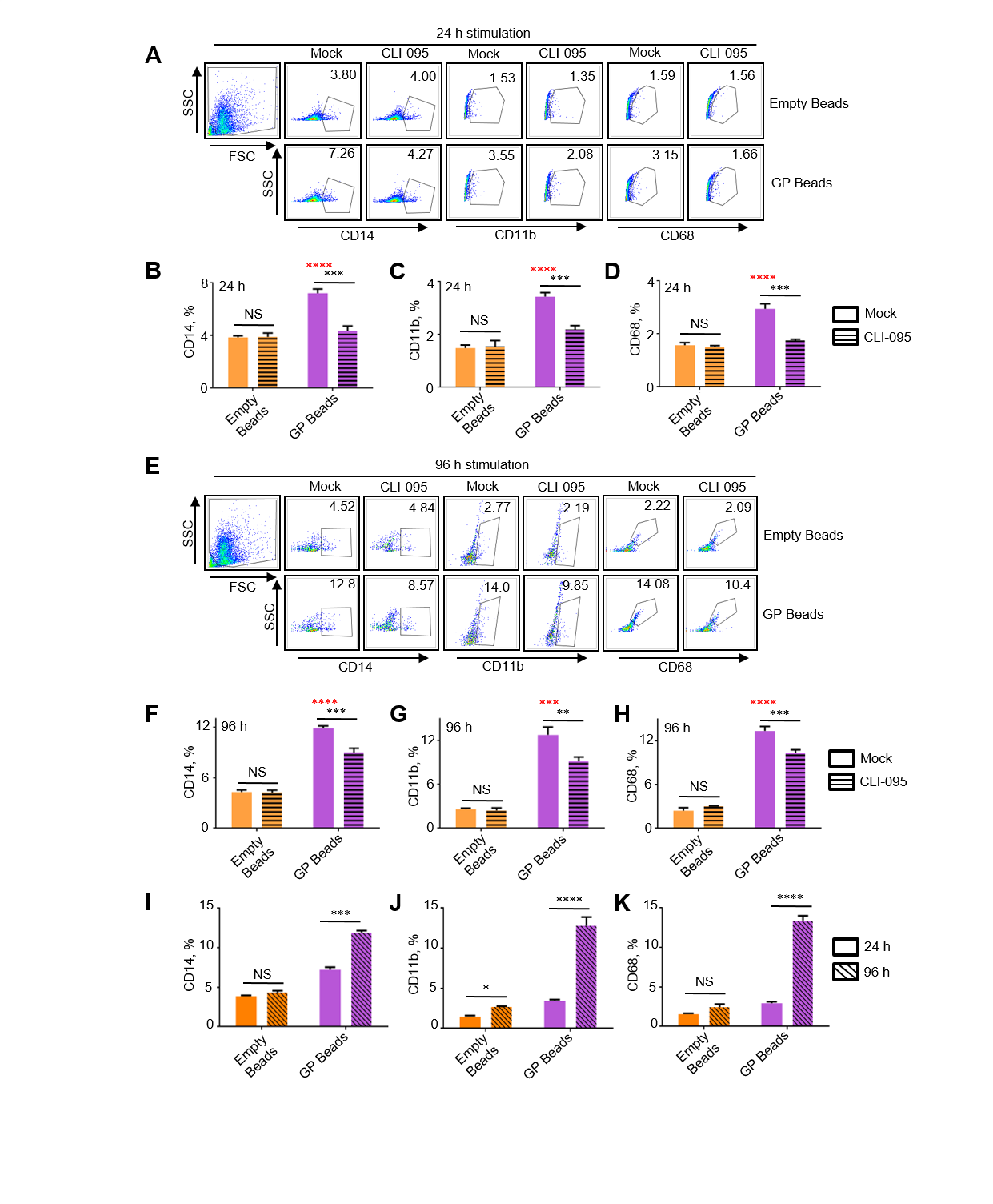

Supplement: S7 Fig — THP-1 cells were treated or mock-treated with CLI-095, cultured with EBOV GP beads or empty beads for 24 h (A-D) or 96 h (E-H) and analyzed by flow cytometry for the indicated markers. Representative flow cytometry data (A, E) with the percentages of the gated populations indicated, and quantitative data showing comparisons of CLI-095-treated and untreated cells (B, C, D, F, G, H), and treatments for 24 h versus 96 h without CLI-095 treatment (I, J, K). Black asterisks, difference to CLI-095-treated cells, red asterisks, difference to mock-stimulated cells. Mean values based on triplicate samples ±SE. * P<0.05, ** P<0.01, *** P<0.001, **** P<0.0001, ns, non-significant. Two-Way ANOVA followed by a Tukey’s Multiple Comparisons Test and multiple T-tests. B-D, F-K, Representative data from two independent experiments. (TIF) [file ppat.1006397.s008.tif]

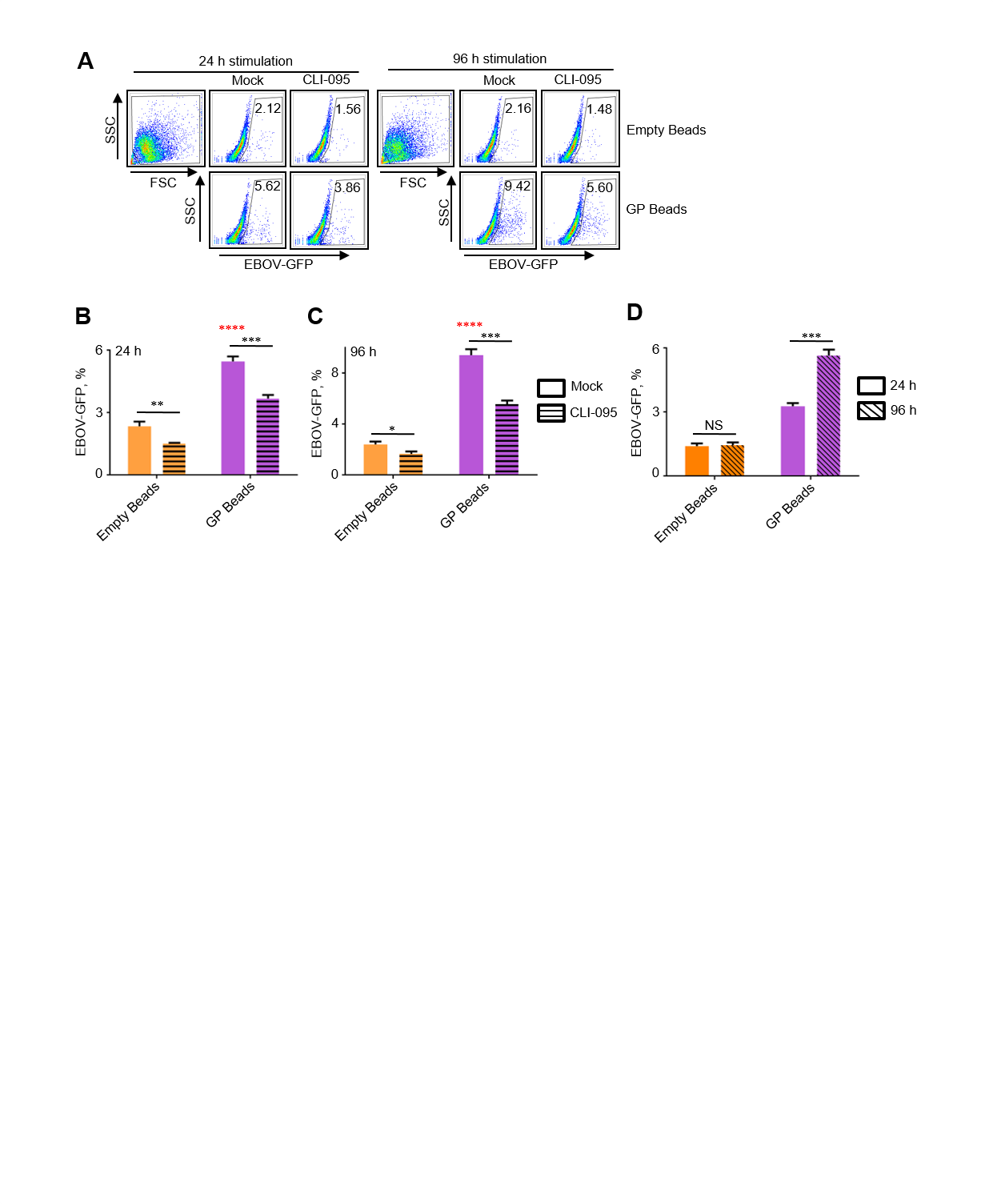

Supplement: S8 Fig — Effects of GP beads on EBOV-GFP infection in THP-1 cells pretreated with CLI-095 analyzed by flow cytometry at 24 and 96 h post infection. A. representative flow cytometry data with the percentages of gated populations indicated. B–D. Percentages of GFP+ cells at 24 h (B), 96 h (C) and comparison of CLI-095-treated samples only and 24 h and 96 h (D). B, C, black asterisks, differences between CLI-095-treated and untreated cells; red asterisks, difference to mock-infected cells. D, asterisks, difference between 24 h and 96 h infection. B-D, Mean values based on three samples per group ±SE with P values calculated using Two-Way ANOVA followed by a Tukey’s Multiple Comparisons Test and multiple T-tests. * P<0.05, ** P<0.01, *** P<0.001. B-D, Representative data from two independent experiments. (TIF) [file ppat.1006397.s009.tif]
